# Interactions of Fungi and Algae from the Greenland Ice Sheet

**DOI:** 10.1007/s00248-022-02033-5

**Published:** 2022-05-24

**Authors:** L. Perini, C. Gostinčar, M. Likar, J. C. Frisvad, R. Kostanjšek, M. Nicholes, C. Williamson, A. M. Anesio, P. Zalar, N. Gunde-Cimerman

**Affiliations:** 1 Department of Biology, Biotechnical Faculty, University of Ljubljana, Jamnikarjeva 101, 1000 Ljubljana, Slovenia; 2 Department of Environmental Science, Aarhus University, 4000 Roskilde, Denmark; 3 Lars Bolund Institute of Regenerative Medicine, BGI-Qingdao, Qingdao, 266555 China; 4 Department of Biotechnology and Biomedicine, Fungal Chemodiversity, Technical University of Denmark, Søltofts Plads, Building 221, 2800 Kgs. Lyngby, Denmark; 5 Bristol Glaciology Centre, School of Geographical Sciences, University of Bristol, Bristol, BS8 1SS UK

**Keywords:** Greenland Ice Sheet, Purpurogallin carboxylic acid-6-O-β-D-glucopyranoside, Purpurogallin carboxylic acid, HPLC, SEM, Light microscopy, *Penicillium anthracinoglaciei*

## Abstract

Heavily pigmented glacier ice algae *Ancylonema nordenskiöldii* and *Ancylonema alaskanum* (Zygnematophyceae, Streptophyta) reduce the bare ice albedo of the Greenland Ice Sheet, amplifying melt from the largest cryospheric contributor to eustatic sea-level rise. Little information is available about glacier ice algae interactions with other microbial communities within the surface ice environment, including fungi, which may be important for sustaining algal bloom development. To address this substantial knowledge gap and investigate the nature of algal-fungal interactions, an ex situ co-cultivation experiment with two species of fungi, recently isolated from the surface of the Greenland Ice Sheet (here proposed new species *Penicillium anthracinoglaciei* Perini, Frisvad and Zalar, Mycobank (MB 835602), and *Articulospora* sp.), and the mixed microbial community dominated by glacier ice algae was performed. The utilization of the dark pigment purpurogallin carboxylic acid-6-O-β-D-glucopyranoside (C_18_H_18_O_12_) by the two fungi was also evaluated in a separate experiment. *P. anthracinoglaciei* was capable of utilizing and converting the pigment to purpurogallin carboxylic acid, possibly using the sugar moiety as a nutrient source. Furthermore, after 3 weeks of incubation in the presence of *P. anthracinoglaciei*, a significantly slower decline in the maximum quantum efficiency (*F*_v_/*F*_m_, inverse proxy of algal stress) in glacier ice algae, compared to other treatments, was evident, suggesting a positive relationship between these species. *Articulospora* sp. did uptake the glycosylated purpurogallin, but did not seem to be involved in its conversion to aglycone derivative. At the end of the incubation experiments and, in conjunction with increased algal mortality, we detected a substantially increasing presence of the zoosporic fungi Chytridiomycota suggesting an important role for them as decomposers or parasites of glacier ice algae.

**Supplementary Information:**

The online version contains supplementary material available at 10.1007/s00248-022-02033-5.

## Introduction

Two species belonging to the Zygnematophyceae, *Ancylonema alaskanum* [1] and *Ancylonema nordenskioeldii*, dominate the surface of the ablation zone of the Greenland Ice Sheet (GrIS) [2–5]. These so-called glacier ice algae represent the most important biological light-absorbing particle in the GrIS ‘Dark Zone’ (western margin) [3, 6–12], contributing an additional 10% of runoff generation during high-melt years [13]. Their impact on surface ice albedo is driven by high production of secondary phenolic pigmentation [8]. High-performance liquid chromatography (HPLC) analysis of the aqueous raw extract of *Ancylonema alaskanum* identified the main pigment as purpurogallin carboxylic acid-6-O-β-D-glucopyranoside (C_18_H_18_O_12_) [14], which is present at 11 times the cellular content of chlorophyll *a* within glacier algal cells [8]. The structure of the compound includes a benzotropolone backbone, hydroxyl and carboxyl groups and a sugar moiety (glucopyranoside) as described by Remias et al. [14]. Primarily, purpurogallin has a photoprotective activity, shielding the chloroplasts from the high irradiance (~ 1700 μmol photos m^−2^ s^−1^) characteristic of the GrIS surface ice environment during summer melt seasons [8], but this phenolic pigment may also play an antimicrobial function [14, 15], or potentially be a carbon source for the heterotrophic community inhabiting the ice surface [16]. However, bacterial biomass and production measured on the surface ice associated with high glacier ice algae abundance are far lower than expected, considering the amount of algal biomass available [2, 16].

Fungi have only recently been identified from the GrIS, with surface ice of the Dark Zone found to harbour a diverse and abundant fungal community [17]. Culture-dependent methods, verified by ergosterol quantification, detected up to 10^5^ CFU/100 ml (colony-forming unit/100 ml) of surface ice [17]. In comparison with other ice habitats, ice with high algal abundance contained a high number of species otherwise known as endophytes and plant pathogens, suggesting that glacier ice algae might act as an environmental filter, shaping the associated fungal diversity and abundance. Overall, two filamentous fungi were noteworthy from a previous study [17]; *Penicillium anthracinoglaciei* was the only *Penicillium* that was also recovered from high algal content ice, and *Articulospora* sp. was observed in close association with glacier algal clumps [17]. *Penicillium* is a cosmopolitan genus, present in Arctic glaciers with high abundance and species diversity [18, 19]. The species *P. anthracinoglaciei* dominated samples from all the environments (surface ice, cryoconite, supraglacial water) collected in 2016 (July–August), and was recovered in 2017 as well, albeit in lower numbers (June–July) [17]. However, its occurrence was not only limited to glacial environments, but the species were also recovered from other habitats, such as refrigerators and museums (Fig. S1). *Articulospora* sp. is an aquatic Ingoldian fungus (from Ingold, the mycologist who discovered the typical habitat of these fungi which grow and sporulate underwater) belonging to the order Helotiales, with a recognized role in carbon dynamics of cryoconite holes in the Arctic (Svalbard) and the potential to influence plant colonisation in glacier forefields [20]. Members of the same genus are recognized as important freshwater decomposers of leaf detritus in Mediterranean regions [21, 22].

In many ecosystems, in addition to playing a role in nutrient cycling, fungi can also act as predators, pathogens and parasites and form symbiotic associations with plants, algae, animals and other organisms [23]. Chytridiomycota, often referred to as chytrids, are a group of saprotrophic and necrotrophic fungi widespread in both aquatic and terrestrial environments [24]. In marine habitats, they have been recognized as important microalgae parasites, able to influence algal bloom dynamics [25]. The presence of chytridiomycetous fungi in a variety of cold habitats has been proven by culture-independent methods [19, 26–29].

Experimental evidence of the interaction between glacier ice algae and other members of the microbial community is currently lacking since glacier ice algae have never been successfully grown in pure culture, despite several attempts [30–32]. In the present study, we focussed on two species of fungi from the surface ice of the GrIS, *Articulospora* sp. and the novel *P. anthracinoglaciei*, and their potential interactions with glacier ice algae. Mixed samples of glacier ice algae and other associated microbes were obtained directly from the GrIS and used during ex situ incubation experiments to investigate potential positive or negative impact of fungi on glacier algal assemblages. The potential for fungal utilization of glacier algal phenolic pigmentation, fungal impacts to algal photophysiology and establishment of putative lichenoid-like relationships was also examined. Finally, to test the possible growth modulation of third microbial partners of the mixed community, antimicrobial compound production was tested for all fungal species incubated during microcosm experiments. The fungal-algal interactions explored here are of particular importance due to the scale of changes occurring in the extremely cold environment of the GrIS, contributing to our understanding of the fungal role in the propagation of glacier algal blooms, which accelerate ice melt.

## Materials and Methods

### Description of Sampling Site and Samples

Sampling for fungal isolation was performed in the southwestern ablation zone of the GrIS in the 2016 (July–August) and 2017 (June–July) summer melt seasons. Glacier ice algae for the co-cultivating experiment were recovered from ice collected ∼60 km east of Kangerlussuaq (67°04′43′′ N 49°20′29′′ W), within the so-called Dark Zone, characterized by extensive glacier algal blooms and consequently, by low albedo [2, 3, 5, 17]. The surface ice of the Dark Zone is dominated by glacier ice algae (*Ancylonema nordenskiöldii* and *Ancylonema alaskanum*), but also contains a mixed microbial community of bacteria, fungi and viruses (e.g. 16, 17, 32). Since in this study we were not able to grow pure algal cultures, when we mention glacier ice algae in the experimental setups, that encompasses the whole microbial community, albeit with a high abundance of glacier ice algae. Surface ice samples of 2 cm depth (5 replicates, approximately 1830 ± 37 ml of ice) and containing high glacier algal abundance (10^4^ cells/ml) were removed using a clean ice saw and transferred into sterile Whirl-Pak® plastic bags. Samples were stored in a dark fridge until transportation to the University of Bristol, UK.

### Isolation of Fungi

For isolation of cultivable fungi, ice was collected and melted aseptically at in situ air temperature (approx. 4 °C). The samples were filtered, and filters were placed onto two enumerations (dichloran rose bengal chloramphenicol agar – DRBC [33], and Reasoner’s 2A agar – R2A [34]) and four different selective agar media (dichloran 18% glycerol – DG-18 [35], Malt Yeast Extract 10% sodium chloride and 12% glucose – MY10-12 [35], synthetic nutrient-poor agar – SNA [36], minimal medium – MM [37]) and incubated at 10 °C as described in Perini et al. [17]. Additionally, samples of melted ice containing a high abundance of glacier ice algae communities were directly inoculated (100 μl) in triplicates on MM, SNA, DRBC, R2A and DG18, and spread with a Drigalski spatula. To prevent bacterial growth, media contained chloramphenicol (50 mg/l) except for R2A. Pure cultures of the identified fungal isolates were deposited in the Ex Culture Collection of the Infrastructural Centre Mycosmo (MRIC UL) at the Department of Biology, Biotechnical Faculty, University of Ljubljana, Slovenia.

### Identification of Fungi

DNA from pure fungal cultures was extracted as described by Perini et al. [17]. For filamentous fungi, a portion of rDNA including the Internal Transcribed Spacer region 1 (ITS1), 5.8S rDNA and ITS region 2 (ITS2) was amplified using ITS5 and ITS4 primers [38]. The ITS nucleotide sequences were determined by Sanger sequencing, performed by Microsynth AG, Switzerland. MUSCLE software [39] implemented in the MEGA5 package [40] was used to align the sequences. The maximum likelihood method implemented in MEGA 5 [40] was used to build phylogenetic trees, which were used to identify the unknown sequences by its proximity to the closest type and reference sequences previously retrieved with the BLAST software from the National Centre for Biotechnology Information (NCBI) GenBank database [41].

**Fig. 1:**
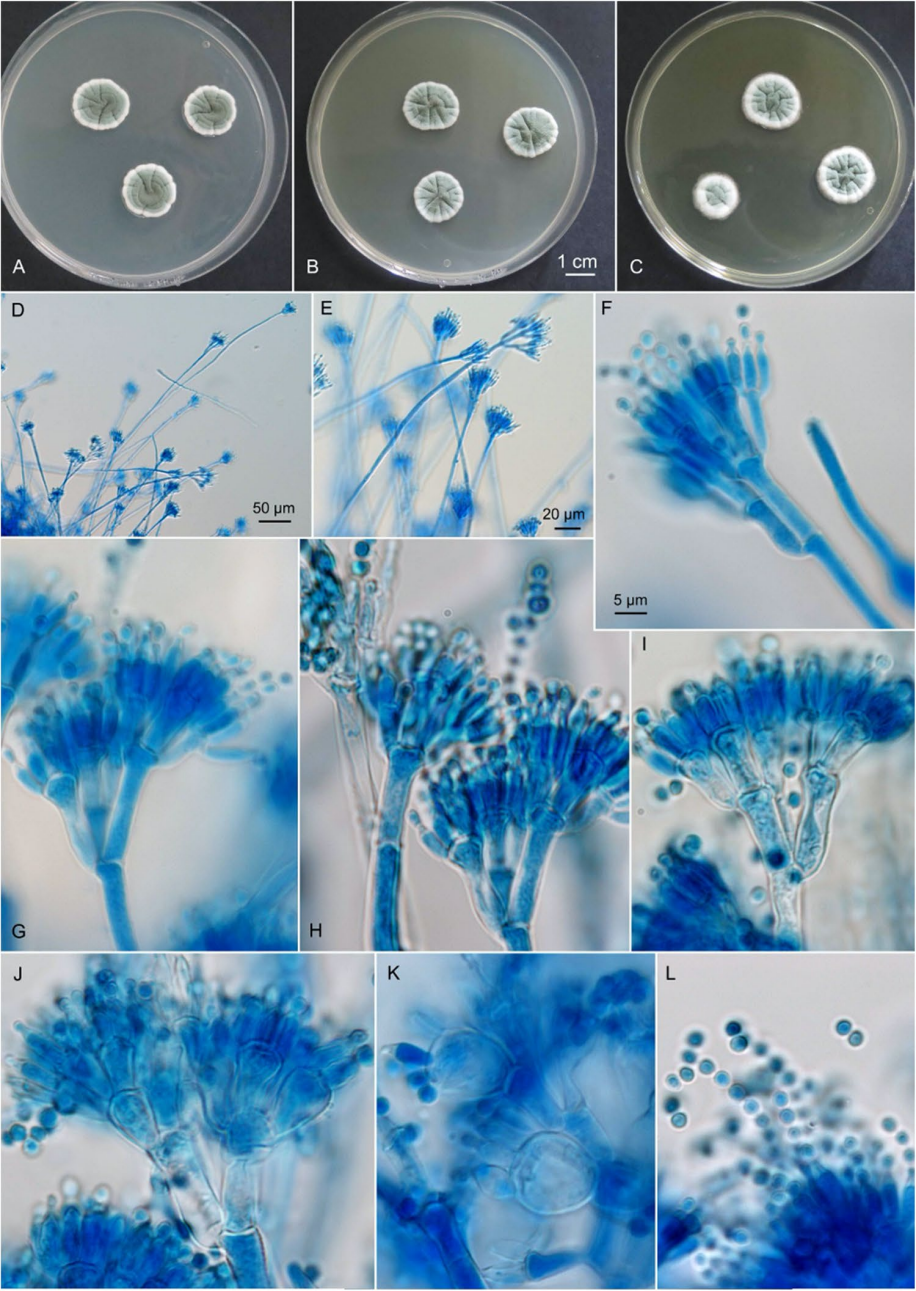
*Penicillium anthracinoglaciei* sp. nov. **A**–**C** Colonies grown at 25 °C for 7 days. **A** CYA. **B** MEA. **C** YES. **D**–**L** Conidiophores and conidia on MEA. **D**–**H** EXF-11443^T^. **I** EXF-10580. **J** EXF-10222. **K** EXF-10226. **L** EXF-11454. Scale bar indicated in **F** valid also for **G**–**L**

A total of 50 different fungal species belonging to 36 genera were isolated and identified from cryoconite, snow, dark and clean supraglacial ice and supraglacial water [17]. Based on cultivable diversity (CFU/100 ml), two of the most abundant filamentous fungi recovered from both seasons were *P. anthracinoglaciei* (42 isolates) and *Articulospora* sp. (59 isolates). Phylogenetic analysis of the combined partial β-tubulin and RNA polymerase 2 (RPB2) genes supported the recognition of a novel species: *P. anthracinoglaciei* with a well-supported clade containing all the isolates from Greenland (Table S1, Fig. S1). Detailed methods and results regarding gene amplification and phylogenetic analysis are provided in the supplementary material. The abundance and repeated isolation of these two fungal species identified them as the best candidates for a co-cultivation experiment to explore the nature of interactions with glacier ice algal communities on the Greenland Ice Sheet. The description of the proposed new species *P. anthracinoglaciei* is provided at the beginning of the “Results” section of the manuscript (Fig. 1). Methods and results for screening of extracellular enzymes and antimicrobial compound production for both fungal species used in the experiment and phylogeny with secondary metabolites profile of *P. anthracinoglaciei* are provided in the supplementary material (Fig. S2, Tables S2–S5).

### Co-cultivation Experiment Setup

Surface ice samples, characterized by a mixed microbial community of bacteria, fungi, viruses and glacier ice algae, were homogenized, transferred into vented sterile cell culture flasks (Corning Inc, USA) and kept at +4 °C in the dark for 3 days prior to the start of the incubation experiment to limit the light stress. Glacier algal maximum quantum efficiency in the dark-adapted state (*F*_v_/*F*_m_, inverse proxy of stress in microalgae) was monitored throughout using pulse-amplitude modulation (PAM) fluorometry as in Williamson et al. [8]. The incubation comprised 5 treatments in triplicates containing varying combinations of glacier ice algae (*Ancylonema nordenskiöldii* and *Ancylonema alaskanum*), and fungi *P. anthracinoglaciei* EXF-11445, and *Articulospora* sp. EXF-13072, isolated from GrIS surface ice (Table 1).

**Table 1:** The five treatments in the incubation experiment. AMBIENT indicates the presence in environmental concentrations, whereas INOCULUM indicates an addition of fungal inoculum at 5% v/v concentration. *Penicillium anthracinoglaciei* spore inoculum concentration was 3 × 10^6^ spores/ml. *Articulospora* sp. biomass inoculum was 1.74 mg dry weight per ml

Treatment		Glacier ice algae	*Articulospora* sp.	*Penicillium anthracinoglaciei*
1	Glacier ice algae (control)	AMBIENT	AMBIENT	AMBIENT
2	Co-culture of glacier ice algae – *Articulospora* sp. EXF-13072	AMBIENT	INOCULUM	-
3	Co-culture of glacier ice algae – *Penicillium anthracinoglaciei* EXF-11445	AMBIENT	-	INOCULUM
4	*Articulospora* sp. (control)	-	INOCULUM	-
5	*Penicillium anthracinoglaciei* (control)	-	-	INOCULUM

Glacier ice algae suspensions were transferred to a 10 L Duran bottle, previously cleaned with 15% hydrogen peroxide and washed with sterile Milli-Q water, wrapped in ice packs to ensure that the suspension remained cold. The Duran bottle was continuously stirred to prevent algal cells from sinking and to ensure a homogenous distribution of glacier ice algae was achieved in subsequent treatments. A sterile glass cylinder was used to divide the suspension randomly between sterile culture flasks (Corning Inc, USA) – final volume of 250 ml. A sterile filter unit containing a 0.45-μm polycarbonate filter (Whatman, England) was used to remove glacier ice algae for fungal control treatments. The filtrate from each unit was divided between cell culture flasks for the respective treatment.

Glacier ice algae *Ancylonema alaskanum* and *Ancylonema nordenskioeldii* were incubated without any fungal culture (treatment 1), or incubated with 5% v/v inoculum of *Articulospora* sp. mycelium (treatment 2), since the fungus does not sporulate in culture, or *P. anthracinoglaciei* spores (treatment 3). Fungal controls in filtered melted ice without the presence of glacier ice algae were also included (*Articulospora* sp. (treatment 4); *P. anthracinoglaciei* (treatment 5)).

Epifluorescence microscopy (Leica DM 2000 epifluorescence microscope) with a Fuchs-Rosenthal haemocytometer (0.2 depth, 1/16 mm^2^, Lancing, UK) was used to quantify initial algal concentrations of 5 × 10^3^ cells/ml across treatments, consistent with their ambient concentration in GrIS surface ice (10^3^–10^4^ cells/ml) [5]. Data available from the GrIS showed that relative ambient frequencies of *Penicillium* and *Articulospora* genera from ITS2 amplicon sequencing results in surface ice are up to 0.3 and 0.06% of all reads, respectively [17]. To verify the interaction of these fungi with glacier algal assemblages, cultures were inoculated with an initial concentration of 3 × 10^6^ spores/ml *P. anthracinoglaciei* across treatments. *Articulospora* sp. biomass inoculum was measured as 1.74 mg dry weight per ml (8.7 mg in 5 ml). Sterile spore suspension solution was also added to treatment 1 to account for any additional nutrients that may be available from this substrate.

The flasks were incubated at 4 °C under a 14/10 h cycle of light-darkness at 181.45 ± 24.5 μmol m^−2^ s^−1^ photosynthetically active radiation (PAR) for 5 months. Light/dark cycles reflected the ambient irradiance regime apparent at our sampling location on the southwestern GrIS. A parallel experiment with continuous dark conditions was performed for all treatments by wrapping flasks with aluminium foil. Culture flasks were mixed every other day and samples randomly redistributed to ensure an even distribution of light across all samples.

Samples for light microscopy, scanning electron microscopy and pulse-amplitude-modulated (PAM) fluorometry were collected in triplicates at five time points; day one of incubations and after 1, 2, 3 weeks, and after 2 and 5 months of incubation.

### Photophysiology

Pulse-amplitude modulation (PAM) fluorometry was used to assess algal photophysiology throughout incubations at each sampling time point for those treatments containing glacier ice algae (up to 5-month incubation). Rapid light response curves (RLCs) were performed following Perkins et al. [42] using a Walz Water-PAM fluorometer (Walz GmBH) consisting of a PAM-control unit with attached cuvette emitter-detector WATER-ED. Samples were dark-adapted for a minimum of 5 min prior to all RLCs, which were performed as 9 incremental light steps of 20 s duration ranging from 0 to 3480 μmol photons m^−2^ s^−1^ PAR, calibrated using a cosine-corrected sensor LI-COR Quantum Sensor (LI-COR Instruments, Lincoln, NE, USA). The maximum quantum efficiency (*F*_v_/*F*_m_) was calculated from minimum (*F*_o_) and maximum (*F*_m_) fluorescence yields in the dark-adapted state as *F*_v_/*F*_m_ = (*F*_m_ – *F*_o_)/*F*_m_. Electron transport through photosystem II (PSII) was calculated from PSII quantum efficiencies (Y[PSII]) in relative units as rETR = Y(PSII) × PAR × 0.5, assuming an equal division of PAR between photosystems I and II. Analysis of RLCs (rETR ~ PAR) followed Perkins et al. (2006), with iterative curve fitting (R, v.3.6.0) and calculation of the relative maximum electron transport rate (rETRmax), maximum light utilization coefficient (*α*) and light saturation coefficient (*E*_k_) following Eilers and Peeters [43].

### Light Microscopy

For the determination of the morphological characteristics and monitoring of interactions between fungi and algae, microscope slides were prepared from incubations and mixed (1:1) with a water solution of 0.1% (w/v) poly-L-lysine. The slides were examined under oil immersion with a BX51 microscope (Olympus, Japan) by differential interference contrast (DIC), at up to ×100 magnification. Digital micrographs were taken with a DP73 digital camera (Olympus, Japan), and analyzed using the cellSens software (Olympus, Japan). Pigmented glacier ice algae were classified as active/alive, whereas unpigmented algae were classified as unactive/dead by manual counting.

### Scanning Electron Microscopy (SEM)

For SEM observation, the samples of co-cultivated algae and fungi were fixed in an equal volume of 0.5% glutaraldehyde + 0.2% formaldehyde at room temperature for up to 2 h. The samples were then washed 3 times with 500 μl of phosphate buffer (7.2 – 7.4 pH) for 7 min, transferred on a polycarbonate membrane (pore size 200 nm) in a filtrating column system and post-fixed in 0.25% OsO_4_ for 30 min. After washing in Milli-Q water, the samples were dehydrated in an EtOH series of ascending concentrations (50%, 70%, 90% and 100%) and acetone for 3 min in each. Acetone was gradually replaced by  hexamethyldisilazane and allowed to air-dry overnight. Filters with dried samples were attached to metal holders, covered with platinum and observed with a JEOL JSM-7500F field-emission scanning electron microscope.

### Quantification and Utilization of the Glycosylated Purpurogallin

To investigate the potential for glacier algal phenolic pigmentation to act as a nutrient source for our two fungal isolates, a complementary incubation experiment was established. Five hundred microliters of glacier algal water-extractable phenolic pigmentation (extracted after Williamson et al. [5] and confirmed here by HPLC to be dominated by purpurogallin carboxylic acid-6-O-β-D-glycopyranoside) was incubated with either *P. anthracinoglaciei* or *Articulospora* mycelium in sterile 5-ml Eppendorf tubes containing 500 μl of sterile minimal medium (MM) 0.5 M without carbon source [37]. Eppendorf tubes were incubated at 15°C for 6 weeks. Fungi were previously grown in malt extract broth (MEB) for 7 days at 15°C. Mycelium were washed with sterile Milli-Q water prior to inoculation.

Pigment concentration was calculated based on the chromatographic peak height (mAU, milli-absorption unit) monitored at 350 nm. Even though peak areas for the detected compounds were also calculated, we preferred to use the peak height of the compounds.

### Confirmation of Identity of Purpurogallin Carboxylic Acid Aglycone by HPLC-MS-MS-DAD

HPLC-MS-MS-DAD analysis was run on an Agilent Q-TOF 6545 using an Agilent Poroshell 120 Phenylhexyl column (2.1mm diameter × 150 mm length, with 1.9 μm particle size) in eluent A consisting of 100% water with 20 mM formic acid and eluent B 100% acetonitrile with 20 mM formic acid, in a gradient running from 10% eluent B to 100% B in 10 min, held at 100% B at 2 min, back to 10% B over 0.1. min, and held at 10% B for 1.9 min. The column temperature was 40°C, and the eluent flow rate was 350 μl/min. The MS range measured was from 100 to 1700 Dalton, with a scan rate of 10 spectra/s, using positive and negative ionization separately. Fragmentation (for MS-MS) was done at 10, 20 and 40 eV [44]. The UV spectrum was compared to the published spectrum [14] and to a standard of purpurogallin (purpurogallin standard purchased from Sigma-Aldrich, as nr. P7380 MSDS), the latter having a slightly different UV spectrum (and retention time) than purpurogallin carboxylic acid. The UV spectra of purpurogallin carboxylic acid and purpurogallin carboxylic acid glycopyranoside were identical (Fig. S3).

### Data Analysis

All statistical analyses of data were performed using R v.3.4.1 [45]. Levene’s test for homogeneity of variance and Shapiro-Wilk's normality test were performed on all data prior to the application of parametric analyses. Consequently, a two-way analysis of variance (ANOVA) was conducted in relation to algal photophysiology for each treatment. Post-hoc Tukey HSD was performed on all significant ANOVA analyses.

## Results

### Description of *Penicillium anthracinoglaciei* Perini, Frisvad and Zalar sp. nov.

*Penicillium anthracinoglaciei* Perini, Frisvad and Zalar sp. nov. Mycobank (MB 835602) (Fig. 1).

In: *Penicillium* subgenus *Penicillium* section *Brevicompacta* [46].

*Etymology*: anthrăcĭnus: “of black colour” (coal black), glăcĭēs, glăcĭēi: “ice”; referring to the origin of this fungus - coal black–coloured ice due to algal bloom causing blackening of Greenland Ice Sheet.

*Morphological description*: colony diameter after 1 week at 25 °C, in mm: CYA: 13–20 (pale yellow reverse); CYAS: 14–20; MEA: 12–15.5; YES: 17–18.5 (green with yellow reverse); CREA: 7–9, moderate growth, acid production. CYA, 5 °C: microcolonies 0–2; CYA, 10 °C: 3–9.5; CYA, 15 °C:13–16.5; CYA, 30 °C, 37 °C: no growth. CYA 15 °C/CYA 25 °C: 0.9 [0.8–1.1], psychrotolerant. Optimum growth temperature: 15–24 °C. CYA/CYAS: 1.1 [0.8–1.4], halotolerant. Conidia *en masse* dark green.

Synnemata or fasciculation: none; sclerotia and ascomata: none; colony texture on CYA: velutinous, radially furrowed; conidium colour on CYA: green to dark green; exudate droplets on CYA: none; reverse colour on CYA: pale yellow; reverse colour on YES: cream yellow; diffusible colour: none; Ehrlich reaction: no or weak violet reaction; conidiophores arising from agar surface; stipes (100–)150–300(–500) × 3–4 (6.5) μm, smooth-walled; penicilli predominantly terverticillate; rami 2–3 per stipe, mostly appressed, (7–)8.5–20 × 2.5–5.5 (–8) μm; metulae 3–4 per ramus, 7–13 (–17) × 3–6 μm, clavate to apically inflated up to 10 μm diam; phialides 4–6 per metula, ampulliform with distinguishable necks, 5.5–11 × 2–4 μm; conidia subglobose to ellipsoidal, 2.5–3.5 × 2–3 μm, walls finely roughened, born in chains that form loose masses (Fig. 1).

*Distribution*: Greenland, Slovenia

*Ecology and habitats*: Greenland, various glacial environments, dust (deposition on museum items), refrigerators.

*Chemotaxonomy* (Table S4): Raistrick phenols, breviones, asperphenamate, quinolactacin 1 and 2, quinolactacin A1, xanthoepocin, mycophenolic acids, orthosporine 1, Alk-747 (an indole alkaloid), Alk-788 (an indole alkaloid), linoleic acid.

*Holotype*: EXF-11443H

*Cultures ex type*: EXF-11443 = IBT 34739, dark ice*,* collected 27.10.2016, Greenland, 67°04′43′′ N 49°20′29′′ W, adjacent to the S6 PROMICE weather station, Laura Perini.

The holotype, EXF-11443H, originated from dark ice in Greenland Ice Sheet, approximately 35 km from the southwestern Ice Sheet margin, 67° 04′43″ N 49° 20′29″ W, adjacent to the S6 PROMICE weather station, in July 2017 by Laura Perini. It is permanently preserved in a metabolically inactive state at the Ex Culture Collection of the Infrastructural Centre Mycosmo (MRIC UL), Slovenia (www.ex-genebank.com) at the Department of Biology, Biotechnical Faculty, University of Ljubljana, Slovenia. Accession numbers of DNA sequences derived from ex-type strain EXF-11443, deposited into the above mentioned Ex Culture Collection: MT080493 (*BenA*), MT080519 (*RPB2*), MT080552 (*CaM*).

Cultures ex type: EXF-11443 = IBT 34739.

The results on the phylogenetic placement of *P. anthracinoglaciei* are provided in the supplementary material (Fig. S1) and detailed results of the comparison of produced secondary metabolites in related species in Table S4.

### Higher Algal F_v_/F_m_ Ratios in *P. anthracinoglaciei* Treatments After 3 Weeks of Incubation

**Fig. 2:**
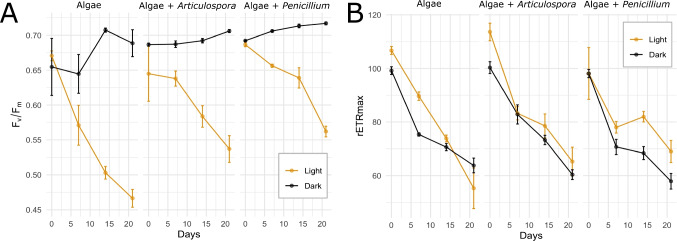
**A** Glacier algae maximum quantum efficiency *F*_v_/*F*_m_ (mean ± standard error, *n* = 3) during the incubation period across both light (orange) and dark (black) treatments. **B** rETRmax (maximum relative electron transport rate) (mean ± standard error, *n* = 3) over the incubation period in each treatment for light (orange) and dark (black) conditions

RLCs confirmed active and healthy glacier algal assemblages at the onset of incubation (day0), with *F*_v_/*F*_m_ averaging 0.67 ± 0.01 and rETRmax 102.7 ± 2.5 across treatments (Fig. 2A, B). To give context to these values, in situ experiments assessing the glacier ice algae photophysiology highlighted that *F*_v_/*F*_m_ ranged around 0.5 and rETRmax ranged between 120 and 160 when incubated under 50% or 0% of irradiance [8]. Values were lower when glacier ice algae were incubated at natural irradiance [8]. After 5 months of laboratory incubation, some degree of variable chlorophyll fluorescence was apparent across treatments with an average *F*_v_/*F*_m_ of 0.51 ± 0.01; however, no measurable electron cycling through photosystem II (PSII) was detectable over RLCs performed using PAM fluorescence (Fig. S5, Table S6). Considering that no active photophysiology was detected after 5 months of incubation, we only present and discuss here the PAM data from up to 3-week incubation, where the glacier ice algae showed signs of active photosynthesis. Across the first 3 weeks of incubation, glacier algal assemblages remained active, though a decrease in their photosynthesis rate (rETRmax) was observed across all treatments (Fig. 2B), likely reflecting the detrimental impacts of laboratory incubations on glacier algal health [30, 31]. Consistent with this, a notable decline was apparent in *F*_v_/*F*_m_ across all incubations under light conditions (Fig. 2A) despite increasing algal abundance (day0 media in light = 4530 ± 1416 cell ml^−1^, 3week-incubation media in light = 8123 ± 2252 cell ml^−1^) (Fig. S4). *F*_v_/*F*_m_ under all dark conditions remained consistent or increased. After 3 weeks of incubation, algae incubated under dark conditions exhibited significantly higher *F*_v_/*F*_m_ as compared to those in the light (algae only: *F*= 59.5, *p*< 0.001; Alg+*Art*: *F*= 58.6, *p*< 0.001; Alg + *Pen*: *F*= 272, *p*< 0.001), possibly due to the lack of light stress compared to the light treatment. Furthermore, *F*_v_/*F*_m_ was significantly higher in glacier ice algae + *Penicillium* treatment in the light as compared to the algal control (*F*= 22.8, *p*< 0.05). On the contrary, *F*_v_/*F*_m_ was not significant for the glacier ice algae + *Articulospora* treatment as compared to the algal control in the light (*F*= 22.8, *p*>0.05) or the dark (*F*= 0.8, *p*>0.05). No significant differences in *α* or *E*_k_ were apparent between treatments of light/dark incubations (Fig. S6 and S7).

### *Penicillium anthracinoglaciei* Had a Beneficial Impact on Glacier Ice Algae Pigmentation After 5-Month Incubation in the Dark

**Fig. 3:**
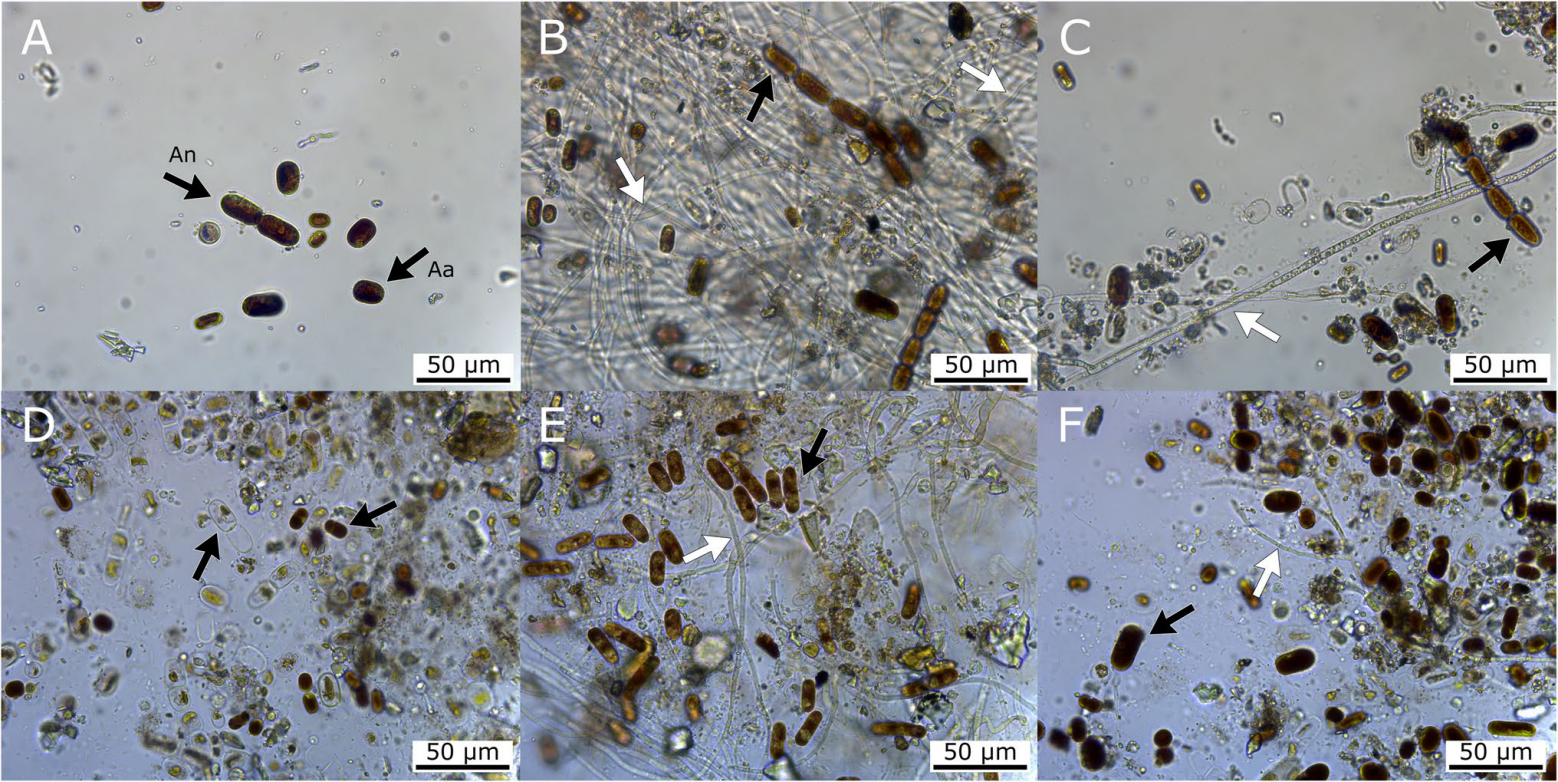
**A**–**C** Light microscopy, light condition, 3-week incubation. **A** algal control; **B** glacier ice algae + *Articulospora*, in this treatment algae are entrapped in the mycelial network; **C** glacier ice algae + *Penicillium anthracinoglaciei*. **D**–**F** Light microscopy, dark condition, 5-month incubation. **D** algal control; **E** glacier ice algae + *Articulospora*; **F** glacier ice algae + *Penicillium anthracinoglaciei*. In the latter treatment, the algae retained their characteristic dark pigmentation. Algal cells: black arrows (Aa, *Ancylonema alaskanum*; An, *Ancylonema nordenskioeldii*); fungal hyphae: white arrows

Microscopy showed that after 3 weeks of incubation, glacier ice algae remained pigmented and formed clumps containing also mineral particles (Fig. 3A). *Articulospora* sp. had densely septated brownish hyphae with many vacuolar inclusions (not shown). No sporulation of *Articulospora* sp. during the experimental incubation was observed. During its growth in co-culture with algae, it formed a net-like hyphal structure that incorporated individual algal cells (Fig. 3B). *P. anthracinoglaciei* spore germination occurred after 1 week of incubation, and after 3 weeks of incubation, its hyphae were found to be in association with algal clumps (Fig. 3C). After 2 months of incubation, *P. anthracinoglaciei* mycelium appeared full of vacuolar inclusions in the fungal control (not shown). Both fungi grew in an extremely low nutrient medium (melted ice) and at a low temperature (+4 °C).

Light microscopy observations of the co-cultivation treatments showed that the phenolic pigment was retained in glacier ice algae under both light and dark conditions after 2 months of incubation. After 5 months, glacier ice algae showed loss of their dominant pigmentation, (Fig. 3D and E) presumably as a consequence of cell mortality except in the *P. anthracinoglaciei* treatment under dark conditions, where glacier ice algae retained their characteristic pigmentation (Fig. 3F). Around 73% of the algal cells were pigmented in the *Penicillium* treatment, compared to 34% of the algal control (Table S7).

**Fig. 4:**
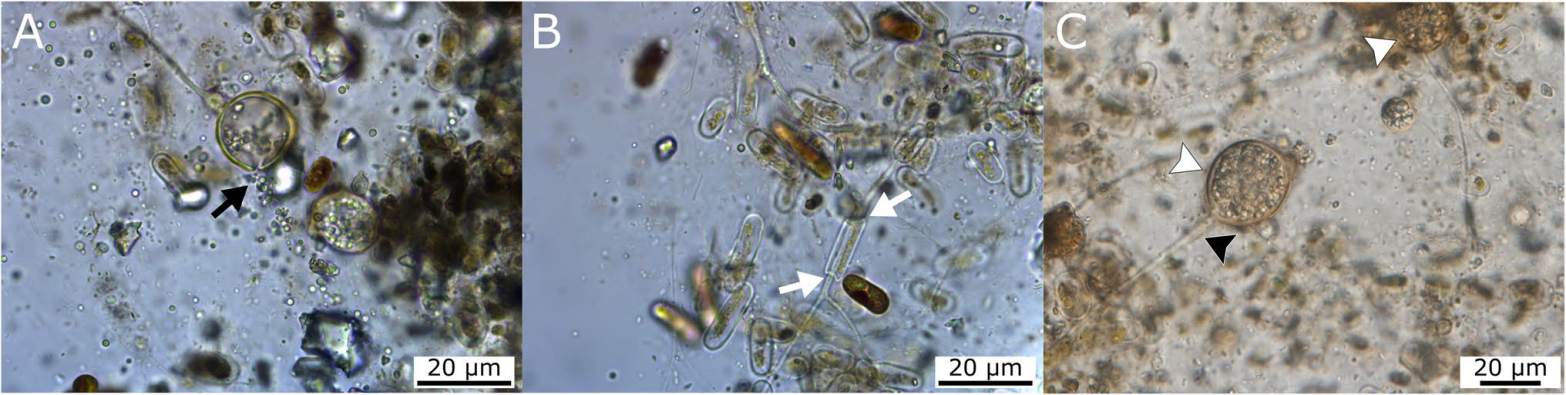
Light microscopy, light condition, 5-month incubation. *Penicillium* treatment. Chytrids releasing zoospores (**A**, black arrow) and interacting with the algae through rhizoids (white arrow) (**B**). Eucarpic-monocentric thalli containing zoospores (white arrowheads) on long pedicel-like rhizoids (black arrowheads) are clearly visible (**C**)

After 5 months of incubation, glacier ice algae in all treatments deteriorated considerably, resulting in the growth and release of a non-targeted group in our experiments, Chytridiomycota fungi and their zoospores (Fig. 4A and B). Eucarpic-monocentric thalli had a spherical shape and were on long pedicel-like rhizoids with extensive branching. Morphological characteristics of the Chytridiomycota are shown in Fig. 4C. All attempts to grow chytridiomycetous fungi thereafter in pure culture were unsuccessful, but since these findings became an interesting feature of the experiments at the end of the incubations, a discussion regarding them will be held.

### *Articulospora* Embedded Glacier Ice Algae on its Hyphal Network

**Fig. 5:**
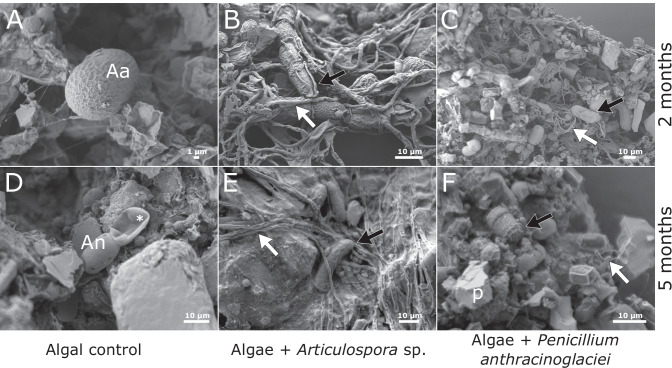
Scanning electron micrographs of algal-fungal co-cultivations. **A**, **D** Algal control (Aa, *Ancylonema alaskanum*; An, *Ancylonema nordenskioeldii*; asterisk - dead cell). Algae (black arrows) incubated with *Articulospora* sp. (**B**, **E**), and *P. anthracinoglaciei* (**C**, **F**) hyphae (white arrows) after 2 months and 5 months of incubation in light condition. **B** *Articulospora* sp. started developing a putative lichenoid structure with embedded glacier ice algae (photobiont) inside the hyphal network. **D**–**F** After 5 months of incubation, cultures appeared more compact after the preparation for SEM (p, mineral particles)

**Fig. 6:**
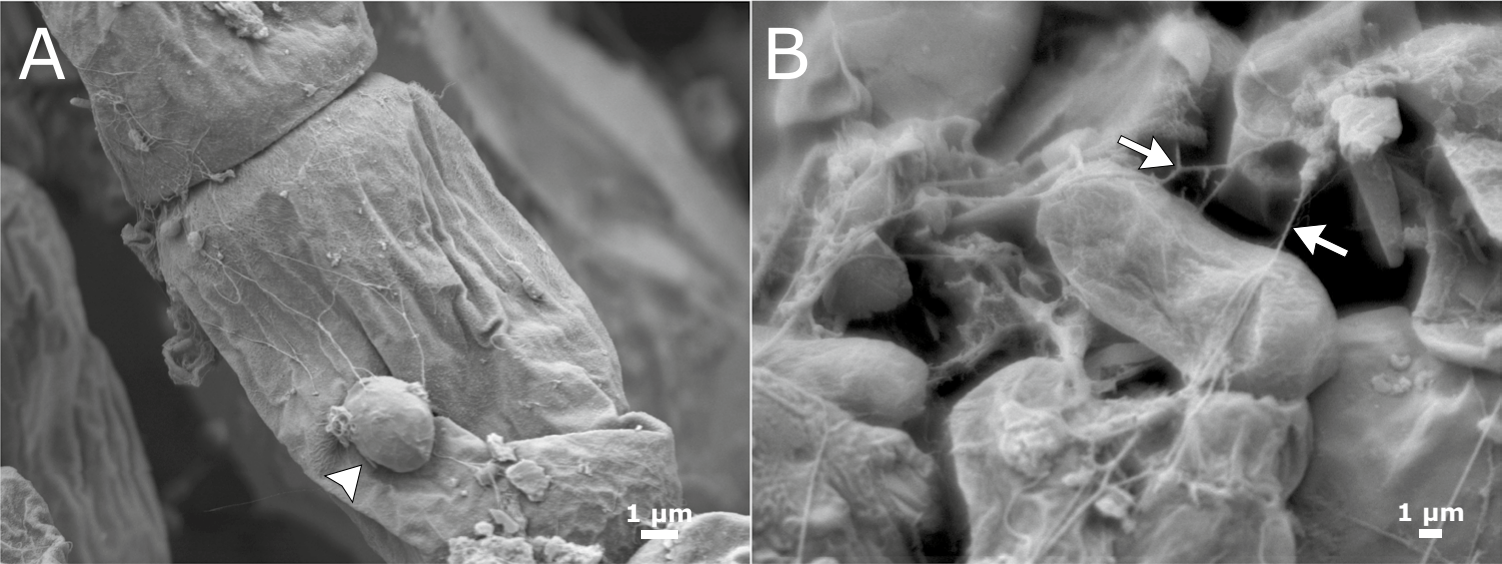
Scanning electron micrograph of an eucarpic-monocentric thallus (white arrowhead) interacting with glacier ice algae (**A**) and their rhizoidal system (white arrows) branching outside of the algal substrate (**B**) under light condition after 5 months of incubation

Scanning electron microscopy revealed preserved outer morphology of both algal species *Ancylonema alaskanum* and *Ancylonema nordenskioeldii* under light and dark conditions after 2 months of incubation (Fig. 5), whilst numerous deflated algae appeared after 5-month incubation time (Fig. 5D), indicating algal mortality. The combination of *Articulospora* sp. and glacier ice algae showed entanglement of the latter in the fibre-like network of *Articulospora* sp. hyphae, with many contact points between the partners after 2 months of incubation (Fig. 5B). After 5 months of incubation, co-cultivated samples were densely packed together (Fig. 5E, F), revealing the presence of chytridiomycetous fungi (Fig. 6A) and their rhizoidal system branching outside the algal substrate (Fig. 6B).

### *Penicillium anthracinoglaciei* Was Able to Interconvert Purpurogallin Glucopyranoside into an Aglycone Derivative

**Fig. 7:**
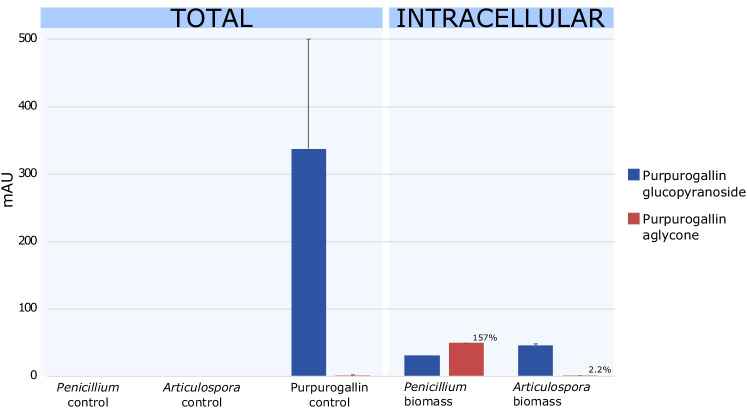
HPLC results as monitored at 350 nm indicated the relative amounts of purpurogallin carboxylic acid-6-O-β-D-glycopyranoside and purpurogallin carboxylic acid aglycone across fungal and pigment controls and treatments after 6 weeks of incubation, both total (extracellular + intracellular) and intracellular. Values were calculated based on the chromatographic peak height and expressed in mAU (mean ± standard error, *n* = 3). The relative percentage on the top of the bars indicates the amount of purpurogallin glycopyranoside transformed into purpurogallin aglycone

The uptake of purpurogallin glucopyranoside by *P. anthracinoglaciei* and *Articulospora* sp. was tested. After 6 weeks of incubation, the negative control (containing no fungal culture) showed in solution (extracellularly) the presence of 337.5 ±162.5 mAU of purpurogallin glucopyranoside and a lower amount of the aglycone derivative (1.1 ±0.9 mAU) (Fig. 7, Table S8). Fungi are preferentially cleaving the sugar moiety for utilization in metabolism and thus driving the interconversion from purpurogallin glucopyranoside to purpurogallin aglycone. Both compounds were identified intracellularly, in the mycelium of both fungi. The aglycone derivative peak in *P. anthracinoglaciei* mycelium was higher (49 ±0 mAU) than purpurogallin glucopyranoside peak (31.25 ±1.75 mAU), which represented a 157 ±9% interconversion within the *Penicillium* treatment. *Articulospora* sp. mycelium contained 46 ±2 mAU of purpurogallin glucopyranoside and 1 ±0 mAU of purpurogallin aglycone, with a lower percentage of interconversion (2.2 ±0.1%).

## Discussion

In this study, we have attempted to map interactions between glacier ice algae (in a mixed microbial community from the ice) and fungal communities isolated from the surface of the GrIS. Whilst fungal concentrations were most likely elevated as compared to ambient concentrations apparent within GrIS surface ice, the purpose of our experiments was not to recreate ambient concentration, but to promote a response between the partners. Fungal concentrations were therefore applied as a treatment to identify the fungal impact on glacier algal assemblages. The microbial community in glacial environments is complex and the interactions between microorganisms may well be more elaborate than implied here. Particularly, besides filamentous fungi that were tested here, abundant species of yeast might have a role in modulating the growth and survival of ice algae.

Our in vitro co-cultivation experiments showed potential associations, with both fungi uptaking the purpurogallin glucopyranoside and with *P. anthracinoglaciei* improving the photosynthetic capabilities of the ice algae, compared to the other treatments. The two fungal species used in the experiments also produced several active compounds, emphasising their ability to regulate the growth of other microbial partners in these associations (Fig. S2, Tables S2–S5). Based on the overall results, we hypothesize different types of fungal-algal interactions occurring in GrIS surface ice.

### *Penicillium*—Positive Association Between Fungi and Glacier Ice Algae

After 3 weeks of incubation, PAM data showed a significantly slower decline in *F*_v_/*F*_m_ (inverse proxy of photosynthetic algal stress) in co-cultures that contained *P. anthracinoglaciei* in comparison with other treatments, indicating that *Penicillium* improved the survivability of glacier ice algae. Whilst this might simply be a result of a shading effect conferred by the fungi, no such positive effect on *F*_v_/*F*_m_ was conferred by *Articulospora* sp. that entrapped algal cells in its hyphal network and would presumably have the same shading impact on glacier ice algae. The beneficial effects of *P. anthracinoglaciei* on algae in vitro might be driven additionally by the production of beneficial secondary metabolites, or mobilization of otherwise unavailable organic or inorganic compounds. For example, several species of penicillia are able to mobilize phosphorus, which has been demonstrated to be a limiting nutrient for glacier ice algae communities [47], making it available to plants, promoting and increasing their growth [48–55]. *P. anthracinoglaciei* showed its ability to produce several secondary metabolites in standard conditions (pure culture, 25 °C) (Table S4). These might affect glacier ice algae directly or indirectly, by modulating the growth or metabolism of a third partner in this mixed community, with a potentially beneficial effect on algal growth. HPLC analysis revealed the presence of mycophenolic acid and its derivatives, xanthoepocin, Raistrick phenols, asperphenamate, quinolactacins and breviones. Mycophenolic acid reportedly proved to inhibit *Candida albicans* and *Staphylococcus aureus*, and several other microorganisms [56]. Xanthoepocin is an anthraquinone dimer with antifungal and antibiotic properties, isolated for the first time from *Penicillium simplicissimum* [57]. Breviones, isolated from *Penicillium* sp. by Tikikawa et al. [58], are bioactive diterpenoid derivatives with potential use in agriculture as herbicides. We hypothesize that *P. anthracinoglaciei* might exert antibacterial activity during active algal growth and photosynthesis. The screening of antimicrobial compounds revealed that *P. anthracinoglaciei* organic extract had a wide antimicrobial spectrum, ranging from fungi to bacteria (Table S5, Fig. S2). It was active both against pathogenic bacteria *Bacillus subtilis* subsp. *spizizenii*, *Staphylococcus aureus* subsp. *aureus* and opportunistic yeast *Candida albicans*, and to environmental isolates of *Micrococcus lactis*, *Sphingomonas* sp., *Cryobacterium psychrotolerans* and *Bacillus* sp.

Light microscopy also highlighted the potential beneficial impact of *P. anthracinoglaciei* on glacier ice algae pigmentation, as evidenced by the prevalence of fully pigmented ("overall" cellular pigmentation) glacier algal cells (73%) following 5 months of incubation in the dark (Fig. 3). In contrast, the proportion of pigmented cells were conspicuously lower (34%) after 5 months of the algal control treatment. Taken together with measurable variable fluorescence at the end of the experiment (average *F*_v_/*F*_m_ = 0.49 ± 0.04; Table S6), these data potentially suggest the entrance on some form of less active stage within our algal assemblages co-incubated with *P. anthracinoglaciei* under dark conditions for a prolonged period. Glacier ice algae are known to over-winter in a vegetative state, presumably as an adaptation to allow rapid onset of metabolism at the start of their relatively short growth season [59]. For example, studies on Alpine glaciers have demonstrated the high freezing tolerance of *Ancylonema alaskanum* and recovery to an active state after ~ 1 week following the end of the winter period and onset of spring ablation [31]. Similarly, active glacier algal assemblages have been observed within shallow sub-surface ice cores at the beginning of the ablation season on the GrIS [16]. In this study, sustained pigmentation of the glacier ice algae during a prolonged (5 months) incubation under complete darkness in the presence of *P. anthracinoglaciei* indicated a direct or indirect beneficial role of this fungus in algal survival. Further research is required to clarify whether glacier ice algae could recover their active physiology after such a prolonged period of darkness.

Incubation of *P. anthracinoglaciei* in a medium with purpurogallin glucopyranoside as the only carbon source resulted in the detection of purpurogallin carboxylic acid aglycone, a compound that exhibited an identical UV spectrum to purpurogallin, but a longer retention time (Fig. S3). These data indicated the ability of *P. anthracinoglaciei* of uptaking the molecule and removing the sugar moiety, possibly utilizing the latter as a nutrient source. This interconversion could occur in the fungus either extracellularly or intracellularly, since both forms of purpurogallin were detected in the medium and mycelium. Since a variety of factors (algal cell burst, death, and release through exudation) can involve the presence of purpurogallin glucopyranoside in the extracellular environment, these data do not contrast with the ability of *Penicillium* to sustain the algal pigmentation after a long period of incubation in the dark. The fungal ability to utilize the glucopyranoside sugar as a nutrient source will be confirmed in a future experiment.

### *Articulospora*—Primitive Lichenoid Association Between Fungi and Glacier Ice Algae

*Articulospora* sp. exhibited a potential lichenoid-like relationship with glacier ice algae. Both light and scanning electron microscopies showed a progressive accumulation of the glacier ice algae, embedded in extracellular polymeric substances (EPS), in *Articulospora* hyphal network, that became particularly distinct after 2 months of incubation. *Articulospora* sp. showed the ability to uptake purpurogallin glucopyranoside in the mycelium; however, its interconversion into purpurogallin aglycone was negligible compared to *Penicillium* in pure culture with purpurogallin glucopyranoside in the medium.

According to the literature, in borderline lichens, two participating species do not form structures resembling a lichen thallus. In these associations, fungal and algal cells grow intertwined with loose contacts observed in both native samples and culture experiments [60, 61]. Because these fungi share strongly oligotrophic environments with green algae, they might take advantage from the presence of the primary producers as a first selective advantage to promote lichenoid-like associations. These loose associations can better proliferate under fluctuating hygroscopic conditions of the subaerial/ice-water native habitat, even when algal cells become more embedded in a thick layer of exopolysaccharides, hampering the formation of contacts. Fungal association with microscopic algae potentially improves their meagre carbon supplies in oligotrophic conditions [62]. We therefore speculate this putative lichenoid-like structure is formed with direct alignment of hyphae and algal cells and many contact points between the partners, possibly with additional contributions of additional symbionts (photosynthetic algae, bacteria and yeasts) [63]. However, after 3 weeks of incubation, the presence of *Articulospora* sp. did not highlight any significant increase in the glacier ice algae *F*_v_/*F*_m_ (inverse proxy of algal stress), either in light or dark treatments, suggesting a neutral effect (neither beneficial nor detrimental) on the algal photophysiology, and/or implying that the incubation conditions may not have been optimal to test the interactions between glacier ice algae and *Articulospora*.

### Chytridiomycota—Parasitic and/or Saprotrophic Fungi

Although this study was not initially designed to investigate the role of Chytridiomycota, a considerable growth of this fungal group was observed by light microscopy in conjunction with increased algal mortality at the termination of our microcosm experiments (after 5 months). Chytridiomycota were only observed sporadically and in smaller numbers during previous sampling time points of the experiment. However, the presence of unidentified Chytridiomycota in dark ice samples from the GrIS was also supported by NGS biodiversity analysis [17], confirming their ubiquity in the GrIS surface ice environments, although none were recovered by cultivation. Moreover, cells of glacier ice algae *A. nordenskioeldii* were found to be colonized by chytridiomycetous fungi in ice samples from Svalbard glaciers [64], hinting at a possible host-specific infection of such glacier ice algae. We intend to address this interesting topic in future research. The length and branching of chytrid rhizoids outside of the algal substrate suggested that a proportion of their nourishment was derived from water dissolved algal or other compounds. Since phenolic pigments might play an antimicrobial function [14], the loss of dark pigmentation apparent during the loss of cell viability may stimulate an overgrowth of the first parasitic and later saprotrophic Chytridiomycota. Chytrids are known for their negative impact on algal biomass in freshwater systems, where they are able to influence algal bloom dynamics, their host gene pool, and phytoplankton size distribution and succession [25]. No information exists to date on the potential top-down controls of glacier algal blooms on the GrIS or indeed across the wider cryosphere [59].

## Conclusions

A study of interactions between glacier ice algae communities and two selected fungal species found in the supraglacial ice of the Greenland Ice Sheet indicated that fungi and algae have different types of interactions.

The interconversion of the pigment purpurogallin glucopyranoside into its aglycone derivative by *P. anthracinoglaciei* suggests its ability of metabolizing the sugar moiety of the pigment, possibly using it as a carbon source. Conversely, *Articulospora* sp. embedded algae into its hyphal network, but failed to provide the algae with any benefits based on the analyses conducted in the experiments. Given the complexity of the systems used in this study, investigation of the potential involvement of additional partners in the fungal-algal interactions would also shed more light on the impact of non-photosynthetic organisms on the GrIS algal blooms.

On the one hand, the increasing presence of Chytridiomycota at the end of the incubation experiment in parallel with higher algal mortality indicates that these zoosporic fungi could act as decomposers or parasites of algal blooms. On the other hand, other fungal species (e.g., *Penicillium anthracinoglaciei* and *Articulospora* sp.) could have a role in sustaining glacier algal blooms during the ablation season or during the over-wintering period, providing various benefits from the mobilization of organic or inorganic compounds, to the secretion of secondary metabolites, physical shielding from light or water currents, induction of dormancy or even modulation of the growth of other microorganisms in putative lichenoid-like structures.

## Supplementary Information

Below is the link to the electronic supplementary material.

Supplementary file1 (DOCX 2.09 MB)

## Data Availability

The dataset generated for this study can be found in GenBank with the following accession numbers: MK460372, MK460374, MK460412, MK460414, MK460417, MK460418, MK460422, MT080468- MT080567.
