# Supplementary material for: Interactions of Fungi and Algae from the Greenland Ice Sheet
Source: Microb Ecol. 2022 May 24;86(1):282–96. doi: 10.1007/s00248-022-02033-5 (PMC10293465; doi:10.1007/s00248-022-02033-5)
Supplement: Supplementary file 1 — Supplementary file1 (DOCX 2.09 MB) [file 248_2022_2033_MOESM1_ESM.docx]

# **SUPPLEMENTARY MATERIAL**

**Article title: Interactions of fungi and algae from the Greenland Ice Sheet**

**Authors:** L. Perini^1,2,*^, C. Gostinčar^1,3^, M. Likar^1^, J.C. Frisvad^4^, R. Kostanjšek^1^, M. Nicholes^5^, C. Williamson^5^, A.M. Anesio^2^, P. Zalar^1^, N. Gunde-Cimerman^1^

^1^ Department of Biology, Biotechnical Faculty, University of Ljubljana, Jamnikarjeva 101, 1000 Ljubljana, Slovenia; ^2^ Department of Environmental Science, Aarhus University, 4000 Roskilde, Denmark; ^3^ Lars Bolund Institute of Regenerative Medicine, BGI-Qingdao, Qingdao 266555, China; ^4^ Department of Biotechnology and Biomedicine, Fungal Chemodiversity, Technical University of Denmark, Søltofts Plads, Building 221, 2800 Kgs. Lyngby, Denmark; ^5^ Bristol Glaciology Centre, School of Geographical Sciences, University of Bristol, BS8 1SS, Bristol, UK

*Corresponding author: Laura Perini, Department of Environmental Science, Aarhus University, 4000 Roskilde, Denmark. E-mail: [laper@envs.au.dk](mailto:laper@envs.au.dk)

**Material and methods**

*Molecular analysis of* Penicillium anthracinoglaciei. DNA was extracted from *P. anthracinoglaciei* strains (Table S1) and stored as described by Van Den Ende and De Hoog (1999). The partial β-tubulin (BenA), the RNA polymerase 2 (RPB2), and the calmodulin (CaM) genes were amplified using the Ben2f - Bt2b (Glass and Donaldson 1995), the RPB2-50F - RPB27cR (Houbraken, Spierenburg, and Frisvad 2012), and the cmd5 - cmd6 (Visagie et al. 2014) primers, respectively. Sequence analysis was performed with a Big Dye Terminator Cycle Sequencing Ready Reaction kit for both strands, and the sequences were aligned with the MT Navigator Software (Applied Biosystems). The resulting sequences of all isolates were aligned using the muscle software implemented in MEGAX package (Kumar et al. 2018). Phylogeny was reconstructed with MrBayes 3.2.6 (Ronquist et al. 2012). Two substitution types of the 4by4 model and gamma distributed rates with a proportion of invariable sites (approximated with 4 categories of gamma distribution) were used for the estimation through 10 million generations (sampling every 100 generation), 2 runs of 15 chains each, heated at temperature 0.2. The data was partitioned into two parts corresponding to BenA and RPB2 sequences and the partition models were treated as unlinked. The final consensus tree was calculated after discarding the first 10% of sampled trees.

Table S1 List of the strains of *Penicillium anthracinoglaciei* considered in the present study: strain collection numbers (EXF), isolation country, year and source, GenBank accession number of the ITS regions of the rRNA sequences, beta-tubulin (BenA), RNA polymerase II (RPB2), and calmodulin (CaM) genes. GrIS: Greenland Ice Sheet. EXF stands for EXtremophilic Fungi.

| **EXF-** | **Isolation glacier and year** | **Isolation habitat** | **GenBank accession number** | | | |
| --- | --- | --- | --- | --- | --- | --- |
|  |  |  | **ITS** | **BenA** | **RPB2** | **CaM** |
| 11216 | GrIS 2016 | Dispersed cryoconite | MK460412 | MT080468 | MT080509 | MT080527 |
| 11218 | GrIS 2016 | Dispersed cryoconite | MK460414 | MT080469 | MT080510 | MT080528 |
| 11220 | GrIS 2016 | Dispersed cryoconite |  | MT080470 |  | MT080529 |
| 11221 | GrIS 2016 | Dispersed cryoconite |  | MT080471 |  | MT080530 |
| 11222 | GrIS 2016 | Dispersed cryoconite |  | MT080472 | MT080511 | MT080531 |
| 11223 | GrIS 2016 | Dispersed cryoconite |  | MT080473 |  | MT080532 |
| 11224 | GrIS 2016 | Dispersed cryoconite |  | MT080474 |  | MT080533 |
| 11226 | GrIS 2016 | Supraglacial water | MK460417 | MT080475 | MT080512 | MT080534 |
| 11227 | GrIS 2016 | Supraglacial water |  | MT080476 |  | MT080535 |
| 11228 | GrIS 2016 | Supraglacial water |  | MT080477 |  | MT080536 |
| 11229 | GrIS 2016 | Supraglacial water | MK460418 | MT080478 |  | MT080537 |
| 11230 | GrIS 2016 | Supraglacial water |  | MT080479 | MT080508 | MT080538 |
| 11231 | GrIS 2016 | Supraglacial water |  | MT080480 |  | MT080539 |
| 11232 | GrIS 2016 | Supraglacial water |  | MT080481 | MT080513 | MT080540 |
| 11233 | GrIS 2016 | Supraglacial water |  | MT080482 | MT080514 | MT080541 |
| 11237 | GrIS 2016 | Cryoconite |  | MT080483 | MT080515 | MT080542 |
| 11239 | GrIS 2016 | Cryoconite |  | MT080484 |  | MT080543 |
| 11240 | GrIS 2016 | Cryoconite |  | MT080485 | MT080516 | MT080544 |
| 11241 | GrIS 2016 | Clear ice | MK460422 | MT080486 | MT080517 | MT080545 |
| 11242 | GrIS 2016 | Clear ice |  | MT080487 |  | MT080546 |
| 11437 | GrIS 2016 | Dispersed cryoconite |  | MT080488 |  | MT080547 |
| 11438 | GrIS 2016 | Dispersed cryoconite |  | MT080489 |  | MT080548 |
| 11439 | GrIS 2016 | Dark ice |  | MT080490 |  | MT080549 |
| 11441 | GrIS 2016 | Dark ice |  | MT080491 | MT080518 | MT080550 |
| 11442 | GrIS 2016 | Dark ice |  | MT080492 |  | MT080551 |
| 11443 | GrIS 2016 | Dark ice |  | MT080493 | MT080519 | MT080552 |
| 11444 | GrIS 2016 | Dark ice |  | MT080494 | MT080520 | MT080553 |
| 11445 | GrIS 2016 | Dark ice |  | MT080495 | MT080521 | MT080554 |
| 11446 | GrIS 2016 | Dark ice |  | MT080496 |  | MT080555 |
| 11447 | GrIS 2016 | Cryoconite |  | MT080497 |  | MT080556 |
| 11448 | GrIS 2016 | Cryoconite |  | MT080498 | MT080522 | MT080557 |
| 11449 | GrIS 2016 | Cryoconite |  | MT080499 |  | MT080558 |
| 11450 | GrIS 2016 | Cryoconite |  | MT080500 |  | MT080559 |
| 11451 | GrIS 2016 | Cryoconite |  | MT080501 | MT080523 | MT080560 |
| 11452 | GrIS 2016 | Clear ice |  | MT080502 |  | MT080561 |
| 11453 | GrIS 2016 | Clear ice |  | MT080503 | MT080524 | MT080562 |
| 11454 | GrIS 2016 | Clear ice |  | MT080504 | MT080525 | MT080563 |
| 11455 | GrIS 2016 | Clear ice |  | MT080505 |  | MT080564 |
| 11456 | GrIS 2016 | Clear ice |  | MT080506 | MT080526 | MT080565 |
| 12422 | GrIS 2017 | Dark ice | MK460374 | MT080507 |  | MT080567 |
| 12428 | GrIS 2017 | Dark ice | MK460372 |  |  | MT080566 |

*Morphological analyses of* Penicillium anthracinoglaciei. To determine the morphological characteristics of the majority of isolated fungi, *Penicillium anthracinoglaciei* isolates have been inoculated as three-point cultures on Czapek Yeast Autolysate agar (CYA), CYA with 5% NaCl (CYAS), Malt Extract Agar (MEA), Yeast Extract Sucrose agar (YES), CREatine sucrose Agar (CREA) and DG-18, and grown for seven days at 25 °C (CYA also at 15 °C and 37 °C) in the dark (Samson and Frisvad 2004). For the determination of micro-morphological characteristics, microscope slides were prepared from MEA and CYA media. Water solution of 60% (v/v) lactic acid with a colour dye was used as the mounting medium. The slides were examined under oil immersion with a BX51 microscope (Olympus, Japan) by differential interference contrast (DIC), at up to 100× magnification. Digital micrographs were taken with DP12 digital camera and analyzed using the DPSOFT 3.2 application software (Olympus, Japan).

*Screening for growth on high concentrations of chaotropic and kosmotropic salts. Penicillium* *anthracinoglaciei* and *Articulospora* sp. were screened for their ability to grow on solid media with high concentrations of kosmotropic (NaCl, KCl and MgSO_4_) and chaotropic (NaBr, MgCl_2_, and CaCl_2_) salts. Fungi were first grown on Malt-Extract Agar (MEA - malt extract 2%, peptone 0.1%, glucose 2%, agar 2%) with no additional salts and incubated at 15 °C for 14 days. Spore suspension for *Penicillium* *anthracinoglaciei* was prepared using spore suspension solution (Tween 80 0.05% (w/v), NaCl 0.9%, agar 0.05%), and had a final optical density of ∼0.8 (600 nm). For non-sporulating *Articulospora* sp., a mycelium suspension was prepared using spore suspension solution. MEA was supplemented with the selected salts at different concentrations (NaCl: 2.0, 2.5, 3.0, 4.0, 5.0 M; KCl: 2.0, 2.5, 3.0, 4.0, 4.5M; MgSO_4_: 2.0, 2.5, 3.0 M; NaBr: 1.5, 2.0, 2.5, 3.0, 3.5, 4.0 M; MgCl_2_: 1.5, 1.6, 1.7, 1.8, 1.9, 2.0, 2.1 M; CaCl_2_: 1.0, 1.2, 1.5, 1.7, 1.9, 2.0 M) as described by Zajc et al. (2014), point inoculated and incubated at 15 °C for up to 6 weeks. Negative controls (non-inoculated medium) for each salinity and salt type were included in the experiments.

*Enzyme production screening in* Articulospora *sp. and* Penicillium anthracinoglaciei. *Articulospora* sp. and *Penicillium anthracinoglaciei* were qualitatively screened for enzymatic activity on solid media. The enzymatic activities tested were fatty acid esterase activity (Tween 80 medium), protease activity (casein and gelatin hydrolysis medium), cellulase production (cellulose medium), pectinolytic activity at pH 5 (pectinase) and pH 7 (pectin lyase) (citrus pectin medium), amylase activity (starch agar), beta-glucosidase activity (aesculin agar), hydrolysis of urea (urease medium), as described by Paterson and Bridge (1994), and tannase activity (tannic acid agar), prepared as described by (Morganna et al. 2017). Fungi were point-inoculated and incubated at 15 °C up to 14 days.

HPLC *– Secondary metabolites profile of* Penicillium anthracinoglaciei*.* Fifty-two isolates belonging to species *Penicillium anthracinoglaciei* (38 isolates), *P. biourgeianum* TYPE (1 isolate), and *Penicillium bialowiezense* TYPE (1 isolate) were analyzed for secondary metabolite production. All tested strains were three-point inoculated onto CYA and YES, packed in perforated sterile plastic bags and incubated at 25 °C in darkness for 8 days. Extracts were performed cutting three agar plugs (6 mm in diameter) for each isolate from different parts of the fungal colonies and added into 2 ml tubes containing 400μl of isopropanol:ethylacetate (1:3 v/v) + 1% of formic acid solution. The tubes were placed into an Ultrasonic bath for 50 min at room temperature. Organic solvents were transferred into new sterile 2ml tubes and dried in a biosafety cabinet overnight. The residues were re-dissolved in 300μl methanol, sonicated for 10 min at room temperature, and then centrifugated for 5 min at 13000 rpm. Supernatant was finally transferred into a clean glass HPLC vial. 1 µl of the methanolic extract was injected into the HPLC and analyzed according to the HPLC method reported by (Nielsen et al. 2017).

*Screening of* Penicillium anthracinoglaciei *and* Articulospora *sp. antimicrobial compounds production. P.* *anthracinoglaciei* and *Articulospora* sp. were screened for the production of antimicrobial compounds. The extracts were prepared from cultures in Erlenmeyer flasks containing 20 ml of Malt-Extract Broth (malt extract 2%, peptone 0.1%, glucose 2%). The flasks were shaken at 150 rpm and 15 °C for 14 days. Fungal secondary metabolites were extracted with chloroform and methanol and distributed to sterile paper discs and the solvent was left to evaporate. The paper discs were added to Nutrient Agar plates previously spread with selected bacterial cultures listed in Table S2. *Staphylococcus aureus* subsp. *aureus* (EXB V128), and *Candida albicans* (EXF-525) were incubated at 37 °C, whereas *Bacillus subtilis* subsp. *spizizenii* (EXB V126), *Pseudomonas aeruginosa* (EXB V129), and *Escherichia coli* (EXB V127) were incubated at 30 °C. All the environmental strains were incubated at 15 °C. Plates were checked for results after 15 h of incubation for the pathogenic strains and after 4 days for the environmental strains.

**Table S2** List of the selected bacterial strains used for the antimicrobial screening, EXF collection number, sampling environment, country and year. CIP: Collection de l’Institut Pasteur. EXB stands for EXtremophilic Bacteria, while EXF stands for EXtremophilic Fungi.

| **Species** | **Culture collection number** | **Environment** | **Locality** | **Sampling Year** |
| --- | --- | --- | --- | --- |
| *Candida albicans* | EXF-525 | Human | Ljubljana, Slovenia | 2009 |
| *Bacillus subtilis* subsp. *spizizenii* | EXB V126 | - | CIP | 2011 |
| *Escherichia coli* | EXB V127 | - | CIP | 2011 |
| *Staphylococcus aureus* subsp. *aureus* | EXB V128 | - | CIP | 2011 |
| *Pseudomonas aeruginosa* | EXB V129 | - | CIP | 2011 |
| *Micrococcus lactis* | EXB L-1922 | Glacial ice | Greenland Ice Sheet | 2016 |
| *Sphingomonas* sp. | EXB L-1972 | Lake ice | Longyearbyen, Svalbard | 2016 |
| *Flavobacterium* sp. | EXB L-1994 | Tap water | Longyearbyen, Svalbard | 2016 |
| *Cryobacterium psychrotolerans* | EXB L-2061 | Dark ice | Greenland Ice Sheet | 2017 |
| *Cryobacterium psychrotolerans* | EXB L-2062 | Dark ice | Greenland Ice Sheet | 2017 |
| *Undibacterium* sp. | EXB L-2063 | Dark ice | Greenland Ice Sheet | 2017 |
| *Pseudomonas* *fluorescens* | EXB L-2644 | Dark ice | Greenland Ice Sheet | 2017 |
| *Pseudomonas* sp. | EXB L-2659 | Dark ice | Greenland Ice Sheet | 2017 |
| *Sphingomonas* sp. | EXB L-2694 | Cloud | Mount Sonnblick, Austria | 2017 |
| *Pseudomonas* sp. | EXB L-2696 | Cryoconite | Greenland Ice Sheet | 2017 |
| *Bacillus* sp. | EXF-12396bac | Glacial ice | Greenland Ice Sheet | 2017 |

**Results**

*Fungal identification and* Penicillium anthracinoglaciei *molecular, morphological and physiological characterization.* During 2016 and 2017 sampling seasons, a total of 50 different fungal species belonging to 36 genera were isolated and identified from cryoconite, snow, dark and clear supraglacial ice, and supraglacial water. Two of the most abundant fungi recovered from both seasons were *Penicillium anthracinoglaciei* and *Articulospora* sp. Phylogenetic analysis of the combined partial β-tubulin (472 bp), and RPB2 (585 bp) genes supported the recognition of a novel species: *Penicillium anthracinoglaciei* with a well-supported clade (*P. bialowiezense*-like sp. 1 group) containing all the isolates from Greenland (Figure S1). Other groups (*P. bialowiezense*-like sp. 2-4) remained unnamed.


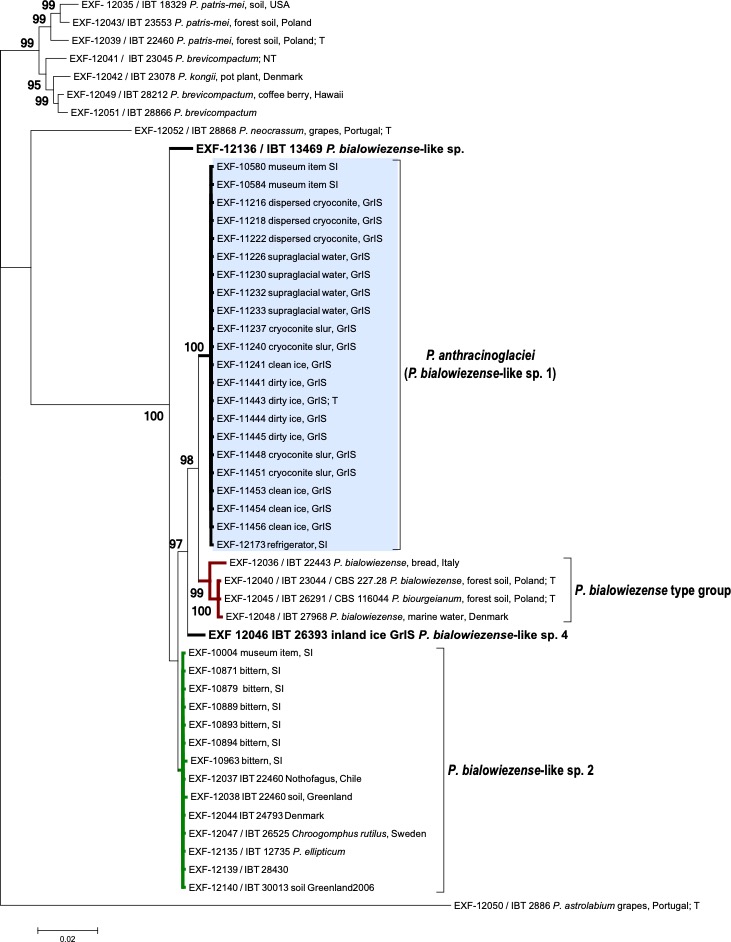


**Figure S1** Phylogenetic tree based on an alignment of the BenA (472 bp) and RPB2 (585 bp) genes rooted with *Penicillium astrolabium* showing the phylogenetic placement of the *P. anthracinoglaciei* within a comprehensive group of closely related species (*P. bialowiezense*-like sp. 1). All phylogenies were reconstructed using Bayesan Markov chain Monte Carlo (MCMC) analyses as implemented in MrBayes 3.2.6. MCMC tree-sampling method values are shown as percentages near tree branches. The scalebar represents number of expected substitutions accumulated per site. Thick lines highlight clusters representing the *P. bialowiezense* type group and new species groups (*P. bialowiezense*-like sp. 1-4).

In total, 42 strains of *P. anthracinoglaciei* were obtained from both sampling seasons (2016 and 2017) (Table S1). During the 2016 season, *P.* *anthracinoglaciei* was the dominant species in all the sampled environments, such as supraglacial water, cryoconite, dark ice and clear ice (Perini *et al.*, 2019a). *P.* *anthracinoglaciei* proved to be extremely oligotrophic (no mycelium development, but long survival in extremely oligotrophic conditions – pure milliQ water, and capable of growth in high salt concentration media (MgSO_4_ 3.0 M, MgCl_2_ 1.5 M, CaCl_2_ 1.5 M, KCl 4.5 M, NaBr 3.0 M, NaCl 3.5 M). Moreover, screening for the enzymatic activity of *P.* *anthracinoglaciei* EXF-11445 highlighted a fatty acid esterase, protease (casein hydrolysis), amylase, beta-glucosidase, urease, and tannase activity (Table S3).

In total 59 strains of *Articulospora* sp. were recovered from the two sampling seasons (2016 and 2017). This species was particularly abundant in cryoconite and dark ice samples at different times of cultivations. Furthermore, *Articulospora* sp. was isolated by direct inoculation of fresh and frozen algal material sampled in 2016, and centrifuged glacier algae sampled in 2017. Enzymatic tests on *Articulospora* EXF-13072 highlighted the ability of this fungus for degradation of fatty acids (fatty acid esterase activity), casein (protease activity), starch (amylase activity), and aesculin (beta-glucosidase activity) (Table S3).

Table S3 Enzymatic activity screening of the fungi *Articulospora* EXF-13072 and *Penicillium anthracinoglaciei* EXF-11445 on solid media. The enzymatic activities tested were fatty acid esterase activity (Tween 80 medium), protease activity (casein and gelatin hydrolysis medium), cellulase production (cellulose medium), pectinolytic activity at pH 5 (pectinase) and pH 7 (pectin lyase) (citrus pectin medium), amylase activity (starch agar), beta-glucosidase activity (aesculin agar), hydrolysis of urea (urease medium), and tannase activity (tannic acid agar).

|  | ***Articulospora* sp.** | ***Penicillium anthracinoglaciei*** |
| --- | --- | --- |
| **MEDIA** | **EXF-13072 (13 days)** | **EXF-11445 (22 days)** |
| TWEEN 80 | + (colony 0.8cm; ring 0.4cm) | + (colony 3.6cm; ring 0.7cm) |
| CASEIN | + (colony 1.1cm; ring 0.5cm) | + (colony 1.7cm; ring 0.6cm) |
| GELATIN | - | - |
| CELLULOSE | - | - |
| PECTINASE | - | - |
| PECTINLIASE | - | - |
| STARCH | + (colony 1cm; ring 0.7cm) | + (colony 1.8cm; ring 1.0cm) |
| AESCULIN | + (colony 3cm; ring 0.5cm) | + (colony 2.7cm; ring 2.2cm) |
| UREASE | - | + (colony 2.2cm; ring 0.3cm) |
| UREASE CONTROL | - | - |
| TANNASE | - (ring 0.8 cm) | + (ring 2 cm) |

Phylogenetic placement of *Penicillium anthracinoglaciei.* Bayesian analysis of aligned, concatenated partial beta tubulin (BenA, 472 bp) and RPB2 (585 bp) sequences of most similar strains from GenBank blast searches and strains deposited in Ex Culture collection of extremophilic fungi resulted in a consensus tree shown in Fig. S1. Our results corroborate other studies (Samson and Frisvad, 2004; Houbraken and Samson, 2011) and indicate that the clade of *P. bialowiezense* and alike taxa is diverse, containing several subclades: the clade containing the type strain EXF-12040 (=CBS 227.28^T^) is well-supported (p.p. 99), as well as a clade containing isolates from Greenland (p.p. 100). Two additional clades are well-supported- the clade containing a single strain EXF-12136 (p.p. 100) and the clade containing single strain EXF-12046. A larger clade containing isolates from Greenland as well as from other various sources was not supported (p.p. 67). Based on these data, we therefore describe strains clustered in clade 1 as *P. anthracinoglaciei*, while the other species we do not describe due to single isolates. Clade *P. bialowiezense* –like sp. 2 is not statistically supported, therefore further analyses are required to elucidate its position.

HPLC – *Secondary metabolite profile of* Penicillium anthracinoglaciei. HPLC analyses revealed that *Penicillium anthracinoglaciei* isolates produce mycophenolic acid, xanthoepocin, Raistrick phenols, asperphenamate, quinolactacins, and breviones (Table S4). The production of secondary metabolites was very consistent between all 38 examined isolates. All isolates produced several derivatives of mycophenolic acid, including mycochromenic acid, mycophenolic acid diol lactone and ethyl mycophenolate. On the one hand, *P. anthracinoglaciei* strains differed from *P. bialowiezense* and *P. biourgeianum* type strains (closest relatives of the described species) in the production of quinolactacin 1 and 2, xanthoepocin, orthosporine 1 and alk-788. On the other hand, unlike *P. anthracinoglaciei* strains, those type strains produced andrastin A. *P. brevicompactum* differs from the three other species in section Brevicompacta by consistently producing brevianamide A and never quinolactacins

**Table S4** Comparison of *P. anthracinoglaciei* in secondary metabolites production with *P. bialowiezense* and P*. biourgeianum* type strains (closest relatives of the described species).

|  | ***Penicillium anthracinoglaciei* (EXF-11445, EXF-11448, EXF-11240)** | ***Penicillium bialowiezense* TYPE (CBS 227.28^T^)** | ***Penicillium biourgeianum* TYPE (CBS 116044^T^)** |
| --- | --- | --- | --- |
| Raistrick phenols | X | X | X |
| Breviones | X | X | X |
| Asperphenamate | X | X | X |
| Quinolactacin 1 and 2 | X |  |  |
| Quinolactacin A1 | X | X | X |
| Xanthoepocin | X |  |  |
| Mycophenolic acids | X | X | X |
| Andrastin A |  | X | X |
| Atlantinone A |  |  |  |
| Orthosporine 1 | X |  |  |
| Brevianamide A |  |  |  |
| Alk-747 | X |  | X |
| Alk-788 | X |  |  |
| Linoleic acid | X | X | X |

*Screening of* Penicillium anthracinoglaciei *and* Articulospora *sp. antimicrobial compounds production.* *Penicillium anthracinoglaciei* had significant antimicrobial activity against one or more of the test microorganisms, generating inhibition halos larger than 11 mm diameter (Table S5 and Figure S2). Species found to be susceptible to secondary metabolites produced by *Penicillium anthracinoglaciei* were the clinically relevant *Bacillus subtilis* subsp. *spizizenii*, *Staphylococcus aureus* subsp. *aureus*, and *Candida albicans*, as well as some environmental strains, including *Micrococcus* sp., *Sphingomonas* sp., *Cryobacterium psychrotolerans* and *Bacillus* sp.

*Articulospora* sp. did not produce any active extractable antimicrobial compounds at the tested condition, either against bacteria or fungi.

Table S5 Antimicrobial compounds test results with zone of inhibition values in mm.

|  |  | **Artic fungal strains (zone of inhibition in mm)** | |
| --- | --- | --- | --- |
|  |  | EXF-11445 *Penicillium* *anthracinoglaciei* | EXF-13072 *Articulospora* sp. |
| **Pathogenic strains** | EXB V126 *Bacillus subtilis* subsp. *spizizenii* | **26** | **-** |
|  | EXB V127 *Escherichia coli* | **-** | **-** |
|  | EXB V128 *Staphylococcus aureus* subsp. *aureus* | **17** | **-** |
|  | EXB V129 *Pseudomonas aeruginosa* | **-** | **-** |
|  | EXF-525 *Candida albicans* | **30** | **-** |
| **Environmental Arctic strains** | EXB L-1922 *Micrococcus* sp. | **22** | **-** |
|  | EXB L-1972 *Sphingomonas* sp. | **11** | **-** |
|  | EXB L-1994 *Flavobacterium* sp. | **-** | **-** |
|  | EXB L-2061 *Cryobacterium psychrotolerans* | **76** | **-** |
|  | EXB L-2062 *Cryobacterium psychrotolerans* | **80** | **-** |
|  | EXB L-2063 *Undibacterium* sp. | **-** | **-** |
|  | EXB L-2644 *Pseudomonas* *fluorescens* | **-** | **-** |
|  | EXB L-2659 *Pseudomonas* sp. | **-** | **-** |
|  | EXB L-2694 *Sphingomonas* sp. | **-** | **-** |
|  | EXB L-2696 *Pseudomonas* sp. | **-** | **-** |
|  | EXF-12396bac *Bacillus* sp. | **19** | **-** |


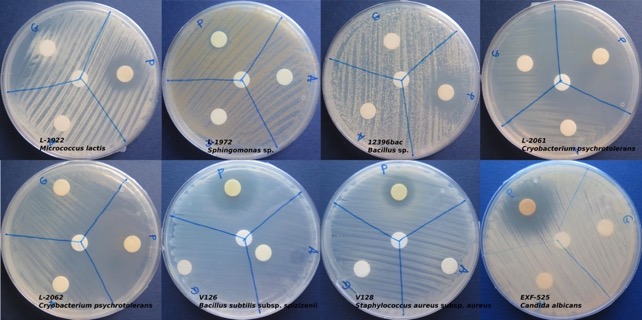


**Figure S2** Bacterial and fungal strains susceptible to the secondary metabolites produced by *Penicillium anthracinoglaciei* (EXF-11445). G -control, A - *Articulospora*, P – *P. anthracinoglaciei*.

**TABLES:**

Table S1. List of the strains of *Penicillium anthracinoglaciei* considered in the present study: strain collection numbers (EXF), isolation country, year and source, GenBank accession number of the ITS regions of the rRNA sequences, beta-tubulin (BenA), RNA polymerase II (RPB2), and calmodulin (CaM) genes. GrIS: Greenland Ice Sheet. EXF stands for EXtremophilic Fungi.

Table S2. List of the selected bacterial strains used for the antimicrobial screening, EXF collection number, sampling environment, country and year. CIP: Collection de l’Institut Pasteur. EXB stands for EXtremophilic Bacteria, while EXF stands for EXtremophilic Fungi.

Table S3. Enzymatic activity screening of the fungi *Articulospora* EXF-13072 and *Penicillium anthracinoglaciei* EXF-11445 on solid media. The enzymatic activities tested were fatty acid esterase activity (Tween 80 medium), protease activity (casein and gelatin hydrolysis medium), cellulase production (cellulose medium), pectinolytic activity at pH 5 (pectinase) and pH 7 (pectin lyase) (citrus pectin medium), amylase activity (starch agar), beta-glucosidase activity (aesculin agar), hydrolysis of urea (urease medium), and tannase activity (tannic acid agar).

Table S4. Comparison of *P. anthracinoglaciei* in secondary metabolites production with *P. bialowiezense* and P*. biourgeianum* type strains (closest relatives of the described species).

Table S5. Antimicrobial compounds test results with zone of inhibition values in mm.

Table S6. Photophysiological parameters determined through the analyses of the glacier algae photoactivity**.** Values are mean ±S.E.M (standard error of the mean). (NA= not applicable; A=algae, Art=*Articulospora*, P=*Penicillium*; L=light, D=dark; 0=time0; 1=one week; 2= 2 weeks; 3=3weeks; 4 = 2 months; 5= 5 months)

Table S7. Percentage of pigmented vs non pigmented algal cells in glacier algae control and algae + *P. anthracinoglaciei* treatments incubated in dark condition after 5 months of incubation (n = 5). Data are normalized.

Table S8. HPLC results monitored at 350 nm. Presence of purpurogallin carboxylic acid-6-O-b-D-glucopyranoside and purpurogallin carboxylic acid aglycone in liquid culture of *P. anthracinoglaciei* and *Articulospora* sp. after 6 weeks of incubation, and their corresponding percentage of conversion. RT = retention time, RI = alkylphenone bracketed retention index. Values are (mAU mean ±standard error).

**FIGURES:**

Figure S1. Phylogenetic tree based on an alignment of the BenA (472 bp) and RPB2 (585 bp) genes rooted with *Penicillium astrolabium* showing the phylogenetic placement of the *P. anthracinoglaciei* within a comprehensive group of closely related species (*P. bialowiezense*-like sp. 1). All phylogenies were reconstructed using Bayesan Markov chain Monte Carlo (MCMC) analyses as implemented in MrBayes 3.2.6. MCMC tree-sampling method values are shown as percentages near tree branches. The scalebar represents number of expected substitutions accumulated per site. Thick lines highlight clusters representing the *P. bialowiezense* type group and new species groups (*P. bialowiezense*-like sp. 1-4).

Figure S2. Bacterial and fungal strains susceptible to the secondary metabolites produced by *Penicillium anthracinoglaciei* (EXF-11445). G -control, A - *Articulospora*, P – *P. anthracinoglaciei*.

Figure S3. HPLC absorption spectra of the pigment purpurogallin carboxylic acid-6-O-β-D-glycopyranoside (A) and purpurogallin carboxylic acid aglycone (B) in the treatment algae + *P. anthracinoglaciei* incubated in the light for 3 weeks, monitored at 350 nm. The absorption is not influenced by the sugar moiety, and therefore the spectra of the two compounds are identical. On the contrary, the retention time is affected by the absence of the sugar, making the compound less polar (RT: 5.20).

Figure S4. Algal abundance expressed as cell/ml (mean ± standard error) during the incubation period across algal control, algae+ *Articulospora* sp., algae + *Penicillium anthracinoglaciei* treatments on day0 (blue), after 3 weeks (yellow), after 2 months (orange) of incubation in light and dark conditions.

Figure S5. Rapid light curves measured with Pulse Amplitude-Modulated fluorimetry at each time point of the incubation to assess changes in algal physiology. Treatment 1 = algal control, treatment 2 = algae + *Articulospora* sp., treatment 3 = algae + *Penicillium anthracinoglaciei*. L = light, D = dark.

Figure S6. E_k_ (light saturation coefficient, mean ± standard error) during the incubation period across both light (orange) and dark (black) treatments.

Figure S7. Alpha (light utilization efficiency, mean ± standard error) during the incubation period across both light (orange) and dark (black) treatments.

Table S6 Photophysiological parameters determined through the analyses of the glacier algae photoactivity. Values are mean ± S.E.M. (NA= not applicable; A=algae, Art=*Articulospora* sp., P=*Penicillium anthracinoglaciei*; L=light, D=dark; 0=time0; 1= one week; 2= two weeks; 3= three weeks; 4 = two months; 5= five months)

| **Sample acronym** | **Light condition** | **Sampling time** | **maximum light utilization efficiency in the dark, Fv/Fm (y, relative units)** | **Maximum relative electron transport rate, rETRm a x (relative units)** | **Light saturation coefficient, Ek (μmol m-2 s-1 PAR)** | **Maximum light use coefficient, α (relative units)** |
| --- | --- | --- | --- | --- | --- | --- |
| Algae | L | 0 | 0.67 (0.0) | 106.74 (1.50) | 375.2 (18.97) | 0.28 (0.01) |
| Algae + *Articulospora* | L | 0 | 0.64 (0.04) | 113.63 (3.25) | 404.7 (9.68) | 0.28 (0.001) |
| Algae + *Penicillium* | L | 0 | 0.69 (0.0) | 98.15 (9.68) | 402.23 (5.86) | 0.33 (0.09) |
| Algae | D | 0 | 0.65 (0.04) | 99.2 (1.43) | 388.46 (16.05) | 0.26 (0.01) |
| Algae + *Articulospora* | D | 0 | 0.69 (0.0) | 100.34 (2.21) | 322.02 (32.65) | 0.32 (0.03) |
| Algae + *Penicillium* | D | 0 | 0.69 (0.0) | 98.11 (1.52) | 366.9 (61.43) | 0.28 (0.04) |
| Algae | L | 1 | 0.57 (0.03) | 89.65 (1.58) | 580.72 (29.92) | 0.15 (0.005) |
| Algae + *Articulospora* | L | 1 | 0.64 (0.01) | 83.24 (2.47) | 404.59 (22.52) | 0.21 (0.02) |
| Algae + *Penicillium* | L | 1 | 0.66 (0.0) | 78.01 (2.16) | 256.58 (42.30) | 0.32 (0.04) |
| Algae | D | 1 | 0.64 (0.03) | 75.34 (0.60) | 380.96 (53.71) | 0.21 (0.03) |
| Algae + *Articulospora* | D | 1 | 0.69 (0.0) | 82.83 (3.57) | 404.21 (71.89) | 0.22 (0.04) |
| Algae + *Penicillium* | D | 1 | 0.7 (0.0) | 70.69 (2.82) | 371.78 (22.51) | 0.35 (0.16) |
| Algae | L | 2 | 0.5 (0.01) | 73.83 (1.26) | 542.56 (73.13) | 0.14 (0.02) |
| Algae + *Articulospora* | L | 2 | 0.59 (0.01) | 78.55 (4.45) | 446 (12.52) | 0.17 (0.006) |
| Algae + *Penicillium* | L | 2 | 0.64 (0.01) | 81.96 (1.98) | 384.67 (35.95) | 0.21 (0.01) |
| Algae | D | 2 | 0.7 (0.0) | 70.6 (1.34) | 328.96 (93.76) | 0.25 (0.06) |
| Algae + *Articulospora* | D | 2 | 0.69 (0.0) | 73.26 (1.75) | 266.41 (30.59) | 0.28 (0.03) |
| Algae + *Penicillium* | D | 2 | 0.71 (0.0) | 68.39 (2.41) | 313.85 (49.25) | 0.23 (0.03) |
| Algae | L | 3 | 0.47 (0.01) | 55.27 (7.57) | 328.99 (11.34) | 0.21 (0.04) |
| Algae + *Articulospora* | L | 3 | 0.54 (0.02) | 65.24 (5.32) | 385.05 (71.17) | 0.18 (0.03) |
| Algae + *Penicillium* | L | 3 | 0.56 (0.01) | 69 (4.09) | 398.19 (42.49) | 0.17 (0.01) |
| Algae | D | 3 | 0.69 (0.02) | 63.79 (2.75) | 246.35 (82.96) | 0.37 (0.17) |
| Algae + *Articulospora* | D | 3 | 0.7 (0.0) | 60.37 (1.81) | 214.7 (2.31) | 0.28 (0.01) |
| Algae + *Penicillium* | D | 3 | 0.71 (0.0) | 57.89 (2.90) | 287.95 (27.35) | 0.2 (0.01) |
| Algae | L | 4 | 0.55 (0.01) | 52.36 (3.25) | 274.44 (38.63) | 0.19 (0.02) |
| Algae + *Articulospora* | L | 4 | 0.55 (0.02) | 45.77 (2.42) | 297.7 (53.68) | 0.16 (0.02) |
| Algae + *Penicillium* | L | 4 | 0.58 (0.01) | 56.93 (13.80) | 317.68 (101.05) | 0.18 (0.01) |
| Algae | D | 4 | 0.61 (0.02) | 58.83 (8.73) | 254.09 (135.82) | 9 (0.12) |
| Algae + *Articulospora* | D | 4 | 0.67 (0.0) | 55.97 (2.03) | 141.11 (9.70) | 0.39 (0.01) |
| Algae + *Penicillium* | D | 4 | 0.55 (0.1) | 64.1 (NA) | 191.12 (NA) | 0.36 (0.02) |
| Algae | L | 5 | 0.56 (0.01) | 18.52 (2.50) | -16.59 (8.04) | -0.18 (1.46) |
| Algae + *Articulospora* | L | 5 | 0.52 (0.0) | 96.9 (48.40) | 1582.71 (711.91) | 0.1 (0.04) |
| Algae + *Penicillium* | L | 5 | 0.52 (0.1) | 131.59 (116.48) | 3360.42 (3415.02) | -0.28 (0.25) |
| Algae | D | 5 | 0.49 (0.1) | 84.41 (75.42) | 14011.34 (13787.93) | 0.21 (0.19) |
| Algae + *Articulospora* | D | 5 | 0.49 (0.04) | 162.77 (44.79) | 28828.96 (12829.98) | 0.01 (0.006) |
| Algae + *Penicillium* | D | 5 | 0.49 (0.04) | 143.94 (92.72) | 24995.53 (18681.17) | 0.03 (0.02) |

Table S7 Percentage of pigmented vs non pigmented algal cells in glacier algae control and algae + *P. anthracinoglaciei* treatments incubated in dark condition after 5 months of incubation (n = 5). Data are normalized.

|  | Algal control R1 | Algal control R2 | Algal control R3 | Algal control R4 | Algal control R5 | Average |
| --- | --- | --- | --- | --- | --- | --- |
| Pigmented cells | 0.46 | 0.41 | 0.32 | 0.30 | 0.22 | 0.34 |
| Non pigmented cells | 0.54 | 0.59 | 0.68 | 0.70 | 0.78 | 0.66 |
|  | Algae + *Pen* R1 | Algae + *Pen* R2 | Algae + *Pen* R3 | Algae + *Pen* R4 | Algae + *Pen* R5 |  |
| Pigmented cells | 0.83 | 0.76 | 0.62 | 0.77 | 0.66 | 0.73 |
| Non pigmented cells | 0.17 | 0.24 | 0.38 | 0.23 | 0.34 | 0.27 |

Table S8 HPLC results monitored at 350 nm. Presence of purpurogallin carboxylic acid-6-O-b-D-glucopyranoside and purpurogallin carboxylic acid aglycone in liquid culture of *P. anthracinoglaciei* and *Articulospora* sp. after 6 weeks of incubation, and their corresponding percentage of conversion. RT = retention time, RI = alkylphenone bracketed retention index. Values are calculated based on the chromatographic peak height and expressed in mAU mean (± standard error).

|  | Purpurogallin carboxylic acid-6-O-b-D-glucopyranoside RT 1.19 RI 554 mAU | Purpurogallin carboxylic acid aglycone RT 5.19 RI 719 mAU | % conversion |
| --- | --- | --- | --- |
| Purpurogallin carboxylic acid-6-O-b-D-glucopyranoside control | 337.5 (162.5) | 1.1 (0.9) | 0.25 (0.15) |
| Purpurogallin carboxylic acid-6-O-b-D-glucopyranoside + *Penicillium anthracinoglaciei* (total material: intracellular + extracellular) | 305 (115) | 22.2 (6.8) | 9.45 (5.75) |
| Purpurogallin carboxylic acid-6-O-b-D-glucopyranoside + *Articulospora* sp. (total material: intracellular + extracellular) | 170.5 (15.5) | 1.5 (0.5) | 0.9 (0.4) |
| *Penicillium anthracinoglaciei* biomass (intracellular) | 31.25 (1.75) | 49 (0) | 157 (9) |
| *Articulospora* sp. biomass (intracellular) | 46 (2) | 1 (0) | 2.2 (0.1) |

**
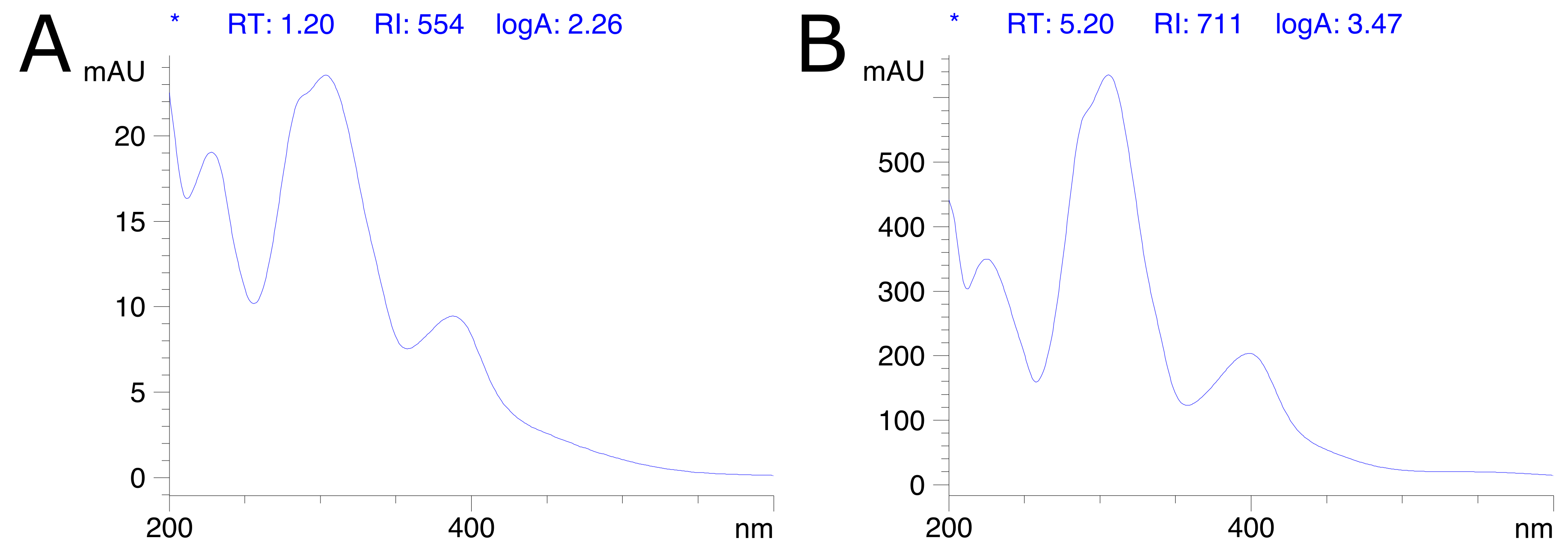
**

Figure S3 HPLC absorption spectra of the pigment purpurogallin carboxylic acid-6-O-β-D-glycopyranoside (A) and purpurogallin carboxylic acid aglycone (B) in the treatment algae + *P. anthracinoglaciei* incubated in the light for 3 weeks, monitored at 350 nm. The absorption is not influenced by the sugar moiety, and therefore the spectra of the two compounds are identical. On the contrary, the retention time is affected by the absence of the sugar, making the compound less polar (RT: 5.20).

**
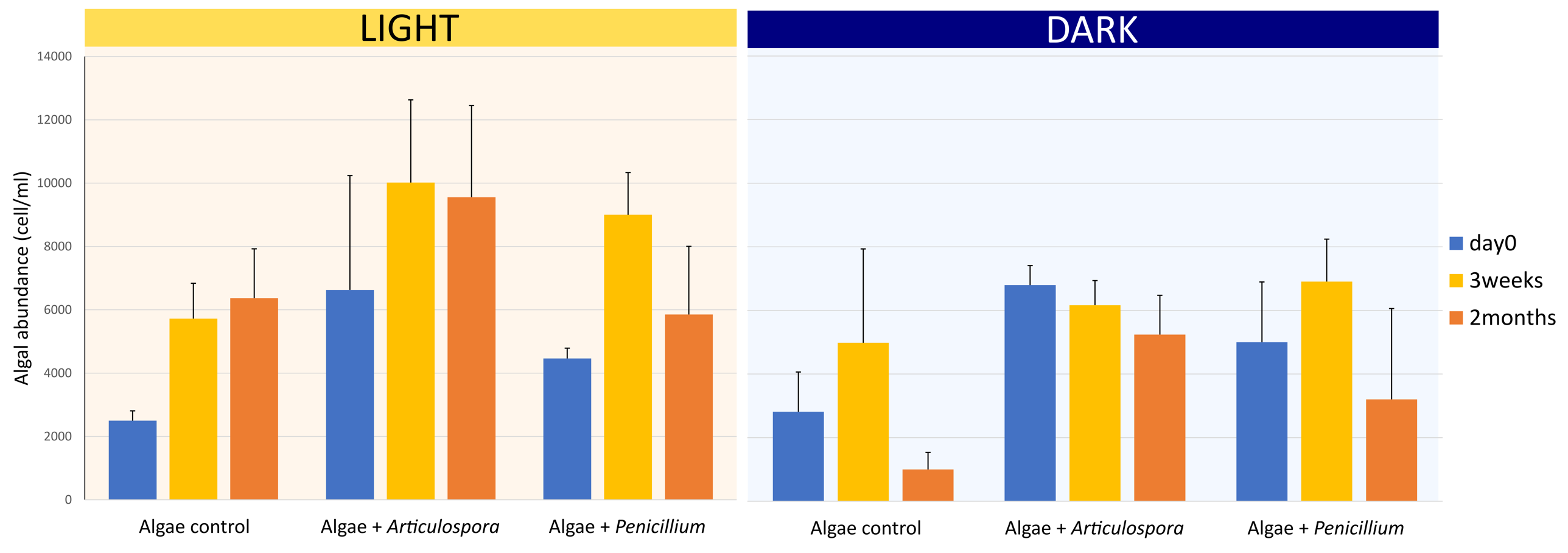
**

**Figure S4** Algal abundance expressed as cell/ml (mean ± standard error) during the incubation period across algal control, algae+ *Articulospora* sp., algae + *Penicillium anthracinoglaciei* treatments on day0 (blue), after 3 weeks (yellow) and after 2 months (orange) of incubation in light and dark conditions.

**
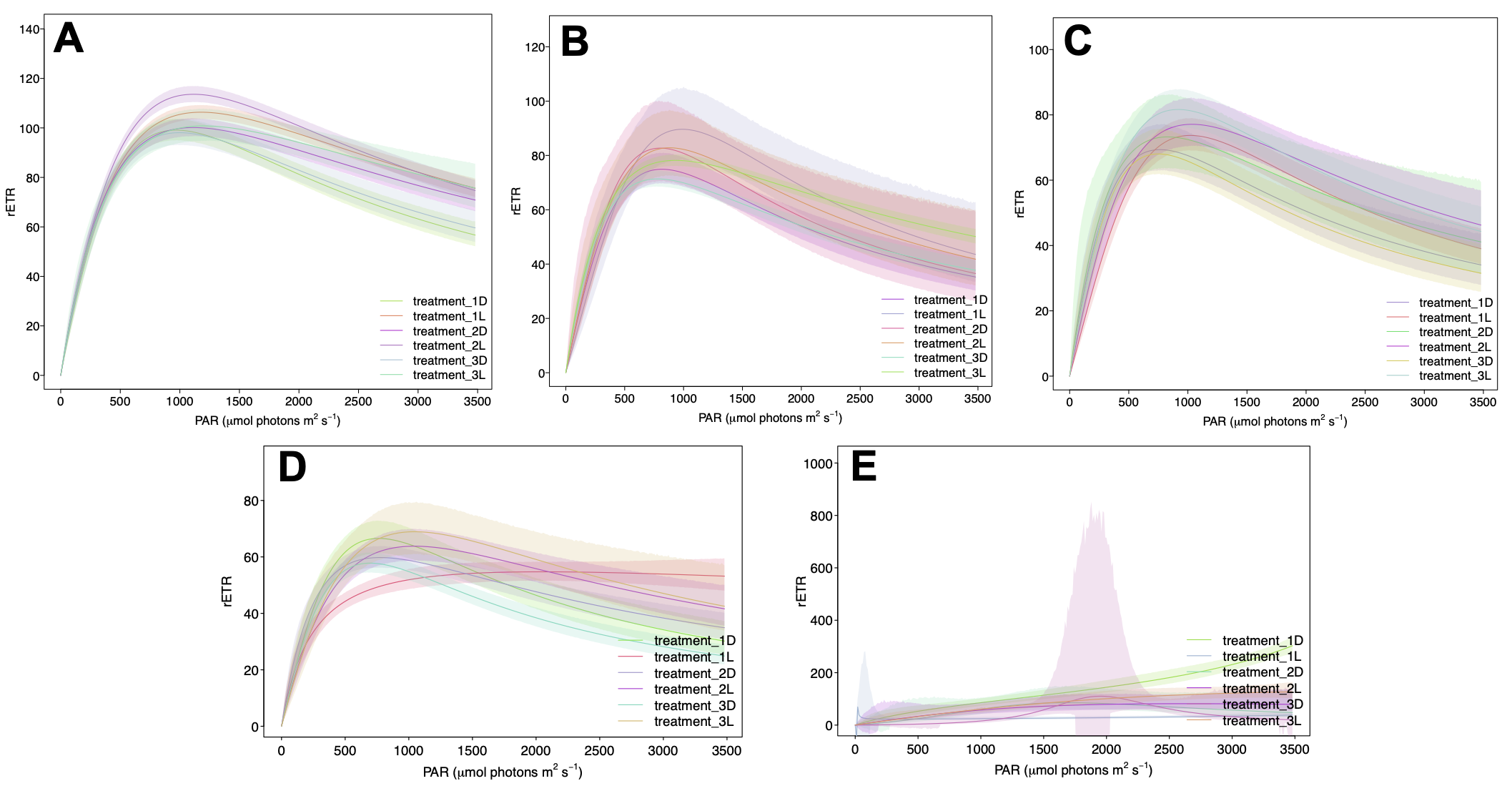
**

**Figure S5** Rapid light curves measured with Pulse Amplitude-Modulated fluorimetry at each time point of the incubation to assess changes in algal physiology. A = time 0, B = 1 week, C = 2 weeks, D = 3 weeks, E = 5 months. Treatment 1 = algal control, treatment 2 = algae + *Articulospora* sp., treatment 3 = algae + *Penicillium anthracinoglaciei*. L = light, D = dark


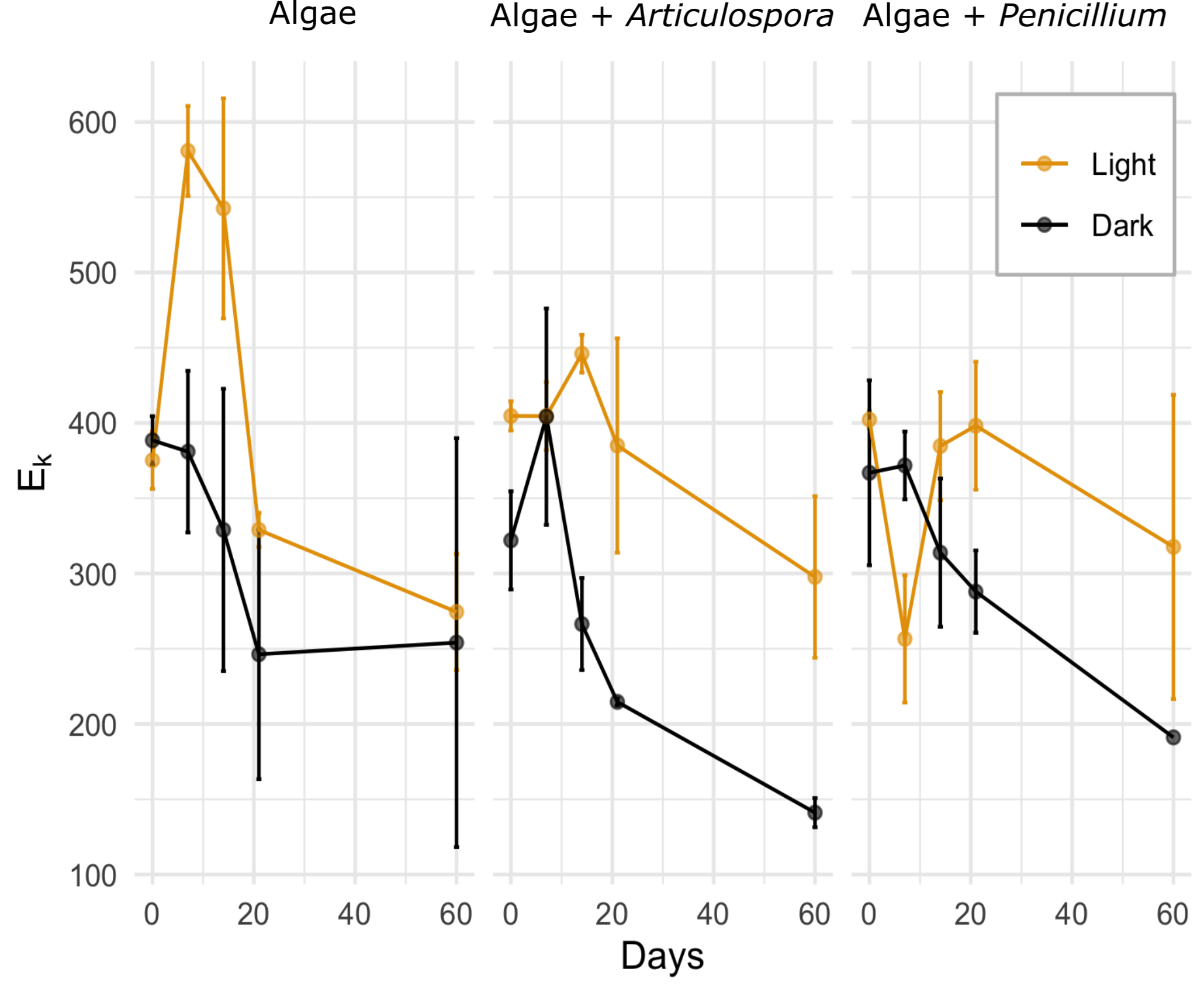


**Figure S6** Ek (mean ± standard error) during the incubation period across both light (orange) and dark (black) treatments.


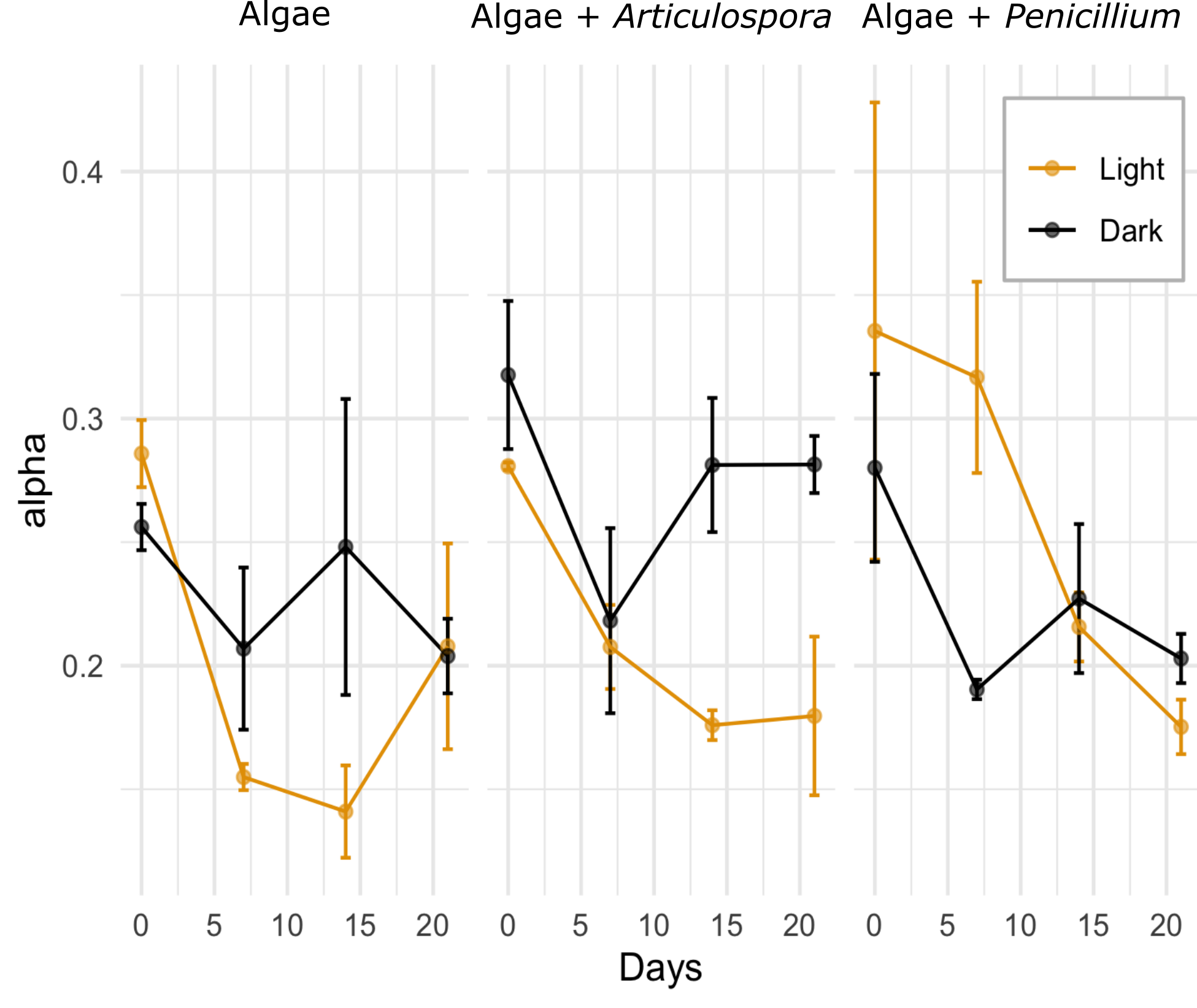


**Figure S7** Alpha (mean ± standard error) during the incubation period across both light (orange) and dark (black) treatments.

**References**

Gerrits Van Den Ende, A. H. G., and G. S. De Hoog. 1999. “Variability and Molecular Diagnostics of the Neurotropic Species Cladophialophora Bantiana.” *Studies in Mycology* 1999(43):151–62.

Glass, N. L., and G. C. Donaldson. 1995. “Development of Primer Sets Designed for Use with the PCR to Amplify Conserved Genes from Filamentous Ascomycetes.” *Applied and Environmental Microbiology* 61(4):1323–30.

Houbraken, J., H. Spierenburg, and J. C. Frisvad. 2012. “Rasamsonia, a New Genus Comprising Thermotolerant and Thermophilic Talaromyces and Geosmithia Species.” *Antonie van Leeuwenhoek, International Journal of General and Molecular Microbiology* 101(2):403–21.

Kumar, Sudhir, Glen Stecher, Michael Li, Christina Knyaz, and Koichiro Tamura. 2018. “MEGA X: Molecular Evolutionary Genetics Analysis across Computing Platforms.” *Molecular Biology and Evolution* 35(6):1547–49.

Morganna, Rayza, Farias Cavalcanti, Pedro Henrique, De Oliveira Ornela, João Atílio Jorge, and Luís Henrique Souza. 2017. “Screening, Selection and Optimization of the Culture Conditions for Tannase Production by Endophytic Fungi Isolated from Caatinga.” *Journal of Applied Biology & Biotechnology* 5(01):1–9.

Nielsen, Jens Christian, Sietske Grijseels, Sylvain Prigent, Boyang Ji, Jacques Dainat, Kristian Fog Nielsen, Jens Christian Frisvad, Mhairi Workman, and Jens Nielsen. 2017. “Global Analysis of Biosynthetic Gene Clusters Reveals Vast Potential of Secondary Metabolite Production in Penicillium Species.” *Nature Microbiology* 2.

Perini, Laura, Cene Gostinčar, Alexandre Magno Anesio, Christopher Williamson, Martyn Tranter, and Nina Gunde-Cimerman. 2019. “Darkening of the Greenland Ice Sheet: Fungal Abundance and Diversity Are Associated With Algal Bloom .” *Frontiers in Microbiology*  10(March):557.

Ronquist, Fredrik, Maxim Teslenko, Paul Van Der Mark, Daniel L. Ayres, Aaron Darling, Sebastian Höhna, Bret Larget, Liang Liu, Marc A. Suchard, and John P. Huelsenbeck. 2012. “Mrbayes 3.2: Efficient Bayesian Phylogenetic Inference and Model Choice across a Large Model Space.” *Systematic Biology* 61(3):539–42.

Samson, R. A., and J. C. Frisvad. 2004. “Polyphasic Taxonomy of *Penicillium* Subgenus *Penicillium*. A Guide to Identification of Food and Air-Borne Terverticillate Penicillia and Their Mycotoxins.” *Studies in Mycology* 49(49):1–173.

Visagie, C. M., J. Houbraken, J. C. Frisvad, S. B. Hong, C. H. W. Klaassen, G. Perrone, K. A. Seifert, J. Varga, T. Yaguchi, and R. A. Samson. 2014. “Identification and Nomenclature of the Genus *Penicillium*.” *Studies in Mycology* 78(1):343–71.

Zajc, Janja, Sašo Džeroski, Dragi Kocev, Aharon Oren, Silva Sonjak, Rok Tkavc, and Nina Gunde-Cimerman. 2014. “Chaophilic or Chaotolerant Fungi: A New Category of Extremophiles?” *Frontiers in Microbiology* 5(DEC):1–15.
